# *ESRG* is critical to maintain the cell survival and self-renewal/pluripotency of hPSCs by collaborating with MCM2 to suppress p53 pathway

**DOI:** 10.7150/ijbs.79095

**Published:** 2023-01-16

**Authors:** Shasha Li, Hui Liu, Weidong Liu, Ning Shi, Ming Zhao, Siyi Wanggou, Weiren Luo, Lei Wang, Bin Zhu, Xiang Zuo, Wen Xie, Cong Zhao, Yao Zhou, Longlong Luo, Xiang Gao, Xingjun Jiang, Caiping Ren

**Affiliations:** 1 NHC Key Laboratory of Carcinogenesis, Department of Neurosurgery, Xiangya Hospital, Central South University, Changsha, Hunan 410008, China.; 2 National Clinical Research Center for Geriatric Disorders, Xiangya Hospital, Central South University, Changsha, Hunan 410008, China.; 3 National Engineering Research Center of Ophthalmology and Optometry, Eye Hospital, Wenzhou Medical University, Wenzhou, 325027, China.; 4 The Key Laboratory of Carcinogenesis and Cancer Invasion of the Chinese Ministry of Education, Cancer Research Institute, School of Basic Medical Science, Central South University, Changsha, Hunan 410078, China.; 5 State Key Laboratory of Toxicology and Medical Countermeasures, Beijing Institute of Pharmacology and Toxicology, Beijing 100039, China.; 6 Department of Neurosurgery, National Clinical Research Center for Geriatric Disorders, Xiangya Hospital, Central South University, Changsha, Hunan 410008, China.; 7 Cancer Research Institute, Shenzhen Third People's Hospital, the Second Affiliated Hospital of Southern University of Science and technology, Shenzhen, Guangdong 518100 China.

**Keywords:** *ESRG*, human pluripotent stem cells, pluripotency, cell survival, OCT4, MCM2, p53

## Abstract

The mechanisms of self-renewal and pluripotency maintenance of human pluripotent stem cells (hPSCs) have not been fully elucidated, especially for the role of those poorly characterized long noncoding RNAs (lncRNAs). *ESRG* is a lncRNA highly expressed in hPSCs, and its functional roles are being extensively explored in the field. Here, we identified that the transcription of *ESRG* can be directly regulated by OCT4, a key self-renewal factor in hPSCs. Knockdown of *ESRG* induces hPSC differentiation, cell cycle arrest, and apoptosis. *ESRG* binds to MCM2, a replication-licensing factor, to sustain its steady-state level and nuclear location, safeguarding error-free DNA replication. Further study showed that *ESRG* knockdown leads to MCM2 abnormalities, resulting in DNA damage and activation of the p53 pathway, ultimately impairs hPSC self-renewal and pluripotency, and induces cell apoptosis. In summary, our study suggests that *ESRG*, as a novel target of OCT4, plays an essential role in maintaining the cell survival and self-renewal/pluripotency of hPSCs in collaboration with MCM2 to suppress p53 signaling. These findings provide critical insights into the mechanisms underlying the maintenance of self-renewal and pluripotency in hPSCs by lncRNAs.

## Introduction

Human pluripotent stem cells (hPSCs), including human embryonic stem cells (hESCs) and induced pluripotent stem cells (hiPSCs), are pluripotent cells with self-renewal capability 1, 2. In-depth study of their self-renewal and differentiation mechanisms is of great significance for understanding the mechanism of human embryonic development, directional induction differentiation and tissue engineering. Their pluripotent state and self-renewal ability are mainly maintained by transcription factors, signaling pathways and epigenetic modifications 3-5. Among them, OCT4, NANOG and SOX2 have been identified as key factors of the transcriptional regulatory network in hPSCs 6-9. These core factors contribute to the hallmark characteristics of hPSCs by activating target genes that encode pluripotency and self-renewal associated proteins and repressing signaling pathways that promote differentiation 10. In hPSCs, OCT4, NANOG and SOX2 co-occupy the promoters of hundreds of protein-encoding genes or long noncoding RNAs (lncRNAs) that are either expressed or repressed in the pluripotent state 11, 12. This pattern suggests that there should be a complex regulatory circuit in which OCT4, NANOG and SOX2 collectively and uniquely regulate downstream targets to control PSC differentiation. However, the downstream effectors of these targets are largely unknown. Additionally, the mechanism by which these “master regulators” of pluripotency control the self-renewal and lineage-specific differentiation of hPSCs also remains to be elucidated.

*ESRG* (also known as *HESRG*), an hPSC-related lncRNA that was first cloned from an expressed sequence tag (EST, BF223023) in our previous study, is highly expressed in hPSCs and is significantly decreased or even completely silenced after differentiation 13. Our previous analysis indicated that *ESRG* is located on human chromosome 3p14.3, which contains four exons and three introns with a full length of 3153 nucleotides. Homologous sequences could only be found in primates, such as* Pan troglodytes and Macaca mulatta,* but not in other species, implying that this gene may be a very specific gene only existing in primate cells 13. No *ESRG* expression was detected in most human cell lines and tissues except in hESCs and several kinds of malignancies, including intracranial germinoma and embryonal carcinoma cells, colorectal cancer cells, and high-grade ovarian serous tumor cells 14-16. In differentiated cells from RA-treated hPSCs and adult tissues, the expression of *ESRG* was tenuous or undetectable. As OCT4 regulates target gene expression by preferentially binding to an octamer sequence (ATTTGCAT), the presence of this octamer at -1,714 to -1,707 bp in the upstream region of the transcription start site of *ESRG* implies that* ESRG* may be directly regulated by OCT4 13.

Recently, *ESRG* has been reported to be useful as a marker to identify early reprogramming cells 17 and as an excellent marker of undifferentiated hPSCs 18. Wang et al. also 19 reported that *ESRG* is required for human-specific pluripotency. These studies suggested that *ESRG* is required for the maintenance of hPSC identity. However, a recent study controverted that *ESRG* is dispensable for human pluripotency 20.

In the current study, we investigated the roles and mechanisms of the regulation and action of *ESRG* in hPSCs and showed that *ESRG* is transcriptionally regulated by OCT4. *ESRG* is involved in the self-renewal and pluripotency maintenance of hPSCs by binding to minichromosome maintenance 2 (MCM2) to sustain its nuclear location and steady-state levels, safeguarding error-free DNA replication. Knockdown of *ESRG* results in DNA damage and activation of the p53 signaling pathway, subsequently promoting hPSC differentiation, cell cycle arrest, and apoptosis. Additionally, our results showed that hPSCs were extremely sensitive to p53 when *ESRG* was knocked down, and once the cells tolerated it, the cells would no longer be affected by p53. These data demonstrated that *ESRG* is an indispensable element in the maintenance of cell survival and self-renewal/pluripotency of hPSCs.

## Materials and methods

### Culture of hPSCs in knockout serum replacement (KSR)-based hPSC medium

Undifferentiated hPSCs [H9 and H1-hESCs (Madison, WI); RC1 and RC2-iPSCs (RC01001, Nuwacell)] were cultured on mitotically inactivated feeder cells (primary mouse embryonic fibroblasts, MEFs) in Dulbecco's Modified Eagle Medium DMEM/F12 (GIBCO) supplemented with 20% serum replacement (Knockout SR) (GIBCO), 1% MEM Non-Essential Amino Acids (GIBCO), 1% GlutaMAX-I (GIBCO), 4 ng/mL basic fibroblast growth factor (bFGF) (Peprotech) and 0.1 mM β-mercaptoethanol (Sigma) 21. The culture medium was refreshed every day, and the cells were conventionally passaged once a week. Cell colonies were loosened from the culture substrate with 1 mg/mL Collagenase Type IV solution (GIBCO, dissolved in DMEM/F12) and gently divided into small pieces with cell scrapers (Becton Dickinson). The hPSC clusters were passaged to a new feeder layer or growth factor reduced Matrigel-(Becton Dickinson)-coated plates at a splitting ratio between 1:6 and 1:10. The presence of the feeder layer complicates mRNA or protein level analysis, so the hPSCs were passaged to Matrigel-coated plates under feeder-free conditions in MEF-conditioned medium (CM) supplemented with 10 mM Y-27632 (Millipore) to maintain pluripotency, as described previously 22. hPSCs cultured on Matrigel-coated plates were passaged as single cells using Accutase solution (Millipore). Cell morphology was recorded using an inverted fluorescence microscope (TE2000U, Nikon).

### Culture of hPSCs in Essential 8 (E8) hPSC medium

hPSCs (H9, H1 RC1 and RC2-iPSCs) were cultured in Essential 8 (E8) medium on plates coated with Matrigel 23. In brief, Matrigel was diluted in DMEM/F12, incubated in dishes for 1 h at room temperature, and then aspirated before replating cells. The hPSCs were passaged every 2-3 days using EDTA dissociation buffer. In brief, the cells were washed twice with DPBS and then incubated with EDTA dissociation buffer for 1-2 min at 37 °C, Then, the EDTA was aspirated and the cells were collected in E8 medium by gentle pipetting and replated at a 1:8-1:12 ratio.

### Culture of other cell lines in DMEM medium

Other cell lines, including 293T cells, MEFs, HeLa cells and COS-7 cells, were grown in DMEM containing 10% FBS (GIBCO) and 1% MEM non-essential amino acids.

### Design and screening of double-stranded siRNAs targeting *ESRG*

Double-stranded siRNAs targeting *ESRG* (si*ESRG*) were designed by different companies or designed using online tools. The siRNAs targeting the *ESRG* RNA sequence could not share significant homology with other genes or sequences in the genome; therefore, homology analysis (www.ncbi.nlm.nih.gov/BLAST/) was performed to avoid off-target effects on other genes or sequences. Finally, 25 sets of si*ESRG* sequences were selected and synthesized by RiboBio Co. (RiboBio). Each designed si*ESRG* sequence corresponded to a different region in the target *ESRG* RNA sequence. To screen for the best-designed siRNA, we transfected the si*ESRG* sequences into hESCs using Lipofectamine RNAiMAX (Invitrogen) 24.

### Lentiviral vector construction, viral production and viral infection

*ESRG* siRNA (si*ESRG*1, si*ESRG*2 and si*ESRG*3) and negative control siRNA (siN0000001, RiboBio) were transformed into shRNA sequences and cloned into the pGV118 vector (GeneChem). The sequences of the effective shRNAs were as follows: si*ESRG* 1: 5'-GGATGGAGCCATAGAAGTT-3', siESRG 2: 5'-GCATGAAAGGGAAGACATA-3'; siESRG 3: 5'-CCATTAAAGGGTCCATCTT-3'. The constructed vectors (pGV118-sh*ESRG*1, pGV118-sh*ESRG*2, pGV118-sh*ESRG*3 and pGV118-shControl) were transfected together with the lentivirus packaging vectors pMDLg/pRRE, pRSV-REV and pCMV-VSV-G (Addgene) into 293T cells using Lipofectamine 2000 (Invitrogen) transfection reagent according to the manufacturer's instructions. The viral supernatant was harvested 48 h after transfection, centrifuged (10 min, 4000 g), filtered using a 0.45 µm Millex-GP filter (Millipore), and then concentrated using a Millipore Centricon-Plus-20 centrifuge filter unit (Millipore). A multiplicity of infection (MOI) of 50 was used to infect hESCs with the lentiviruses 25, 26. Cells were dissociated with Accutase solution and seeded in CM supplemented with 10 mM Y-27632 on Matrigel-coated six-well plates, together with the generated lentiviruses and 8 μg/mL of polybrene (Sigma) for viral infection. To confirm the knockdown efficiency of *ESRG*, cells were harvested for qPCR analysis 5 days after viral infection.

### Establishment of a tetracycline- (Tet-) inducible shRNA system

To establish a stable hESC line that constitutively expressed the TetR protein, H9 hESCs were transfected with the pCAG-TetRnls vector (a kind gift from Peter W. Andrews) using FuGENE HD Transfection Reagent (Promega) and then selected using 2 μg/mL puromycin (Sigma) 48 h after transfection. Cell clones (H9-TetR cells) that highly expressed TetR protein were selected, expanded, and used in subsequent studies.

According to the pSUPER RNAi system user manual (Oligoengine), OCT4 siRNA with the target sequence GGATGTGGTCCGAGTGTGGT, *ESRG* siRNA with the target sequence GGATGGAGCCATAGAAGTT and β2 M siRNA (negative control) with the target sequence GGACTGGTCTTTCTATCTCT were designed as shRNAs using Oligoengine 2.0 and then cloned into the pSUPERIOR.retro.neo+GFP vector (Oligoengine) 27. Subsequently, H9-TetR cells were transfected with the constructed vectors pSUPERIOR-sh*OCT4*, pSUPERIOR-sh*ESRG* or pSUPERIOR-shβ2 M using FuGENE HD and selected with 300 μg/mL G418 (Sigma). Once single, stable transfectants (H9-TetR-sh*OCT4*/H9-TetR-sh*ESRG* cells) were generated, they were isolated, cultured on Matrigel-coated plates in CM, and assessed by qPCR for the efficiency of gene knockdown following treatment with doxycycline (Dox, Sigma).

### Adenovirus vector construction and transfection

*ESRG* expression adenovirus and control adenovirus were synthesized by Vigenebio (Shandong, China). According to the actual situation and MOI value of the experiment, the adenovirus stock solution was diluted in the culture medium and then added to the target cells. After being mixed, the cells were incubated overnight in an incubator at 37 °C and 5% CO_2_. After 12 h of virus infection, the culture medium was changed and tested 48 h later.

### Construction of the *ESRG* Pr-tdTomato reporter hPSC line

We constructed an *ESRG* Pr-tdTomato reporter hPSC line using gene editing techniques to insert fusion sequences of the *ESRG* promoter and tdTomato fluorescent protein gene into the AAVS1 human safe harbor locus as previously described 28.

### Construction of truncated, antisense vector and domain vector

The truncation and antisense sequences of *ESRG* were cloned into the pcDNA3.1(+) vector. The Flag-MCM2 domain (MCM2 full-length, MCM2-N, MCM2-C, MCM2-N1, MCM2-N2, and MCM2-N3) expression plasmids were cloned into the PB212 (1 BsiWI)-Puro vector.

### RNA isolation and qPCR

Total cellular RNA was isolated with TRIzol reagent (Invitrogen) as described previously 29, followed by treatment with deoxyribonuclease I (DNase I, Promega) to remove any contaminating genomic DNA. First-strand cDNA was synthesized using 1 μg of total RNA and a RevertAid First Strand cDNA Synthesis Kit (Fermentas). qPCR analysis was then performed using FastStart Essential DNA Green Master Mix (Roche) and an MJ Mini Personal Thermal Cycler (Bio-Rad). The threshold cycle (Ct) values of each sample were used in the post-PCR data analysis and normalized against endogenous *GAPDH*. qPCR was conducted in triplicate for each sample. The primer sequences for the qPCR are listed in Table S3.

### Isolation of RNA and protein from the nucleus and cytoplasm

The extraction of RNA and protein was performed according to the PARIS Kit (Invitrogen, AM1921). Briefly, 10^7^ cells were collected and disrupted in 100-500 µL ice-cold Cell Fractionation Buffer and centrifuged for 5 min at 4 °C and 500 g after being incubated on ice for 5-10 min. Then, the cytoplasmic fraction was carefully aspirated away from the nuclear pellet, and the nuclear pellet was lysed in Cell Disruption Buffer. Finally, the lysate was divided for RNA isolation and protein analysis.

### AP staining

AP staining was performed using a SIGMAFAST BCIP/NBT tablet (Sigma) following the manufacturer's instructions. Briefly, cells were washed twice with PBS and stained with SIGMAFAST BCIP/NBT substrate solution in the dark for 5-10 min. Then, the substrate solution was removed when sufficient color had developed 22. After staining, the cells were washed with PBS, and bright-field images were taken using an inverted fluorescence microscope (TE2000U, Nikon).

### Western blot analysis

Western blot analysis was performed as previously described 30, 31. Briefly, cells were washed twice with PBS and lysed in M-PER Mammalian Protein Extraction Reagent (Thermo) containing 1×Halt Protease Inhibitor Cocktail (Thermo) and PhosSTOP phosphatase inhibitors (Roche). Equal amounts (20-40 μg) of total protein were boiled, electrophoresed on 12% SDS-polyacrylamide gels and electroblotted onto PVDF membranes (Millipore). Membranes were blocked for 1 h with 5% fat-free milk solution or 5% BSA, probed with primary antibodies overnight at 4 °C, and then incubated with secondary antibodies at room temperature for 2 h. The blot was visualized using enhanced chemiluminescence (ECL, Thermo). Images were recorded using the Molecular Imager ChemiDoc XRS+ System (Bio-Rad) and analyzed with Quantity One 1-D analysis software (Bio-Rad). The antibodies used in this study are listed in Table S4.

### Cell cycle analysis

hPSCs were carefully dissociated into single-cell suspensions using Accutase solution, washed twice with PBS, and then fixed overnight with cold 70% ethanol. The fixed cells were washed twice with PBS, followed by RNase (Sigma, 100 µg/mL) treatment and propidium iodide (Sigma, 50 µg/mL) staining for 30 min at 37 °C. Approximately 1×10^6^ cells were analyzed using a FACSCanto II (Becton Dickinson) to determine the cell cycle distribution pattern. The percentages of cells in G1, S and G2/M phases of the cell cycle were analyzed using ModFit 4.0 software (Verity Software House).

### Cell proliferation assays

Cell proliferation was assessed using a Cell Counting Kit-8 (CCK-8, Vazyme). Briefly, cells were seeded as single cells in the wells of a 96-well plate coated with Matrigel and transfected with si*ESRG* or control siRNA. At different time points after siRNA treatment, the cells were incubated for 4 h with CCK-8 at 37 °C. The absorbance of the formazan dye generated by the dehydrogenase activity in living cells was measured using a PARADIGM Detection Platform (Beckman Coulter) at a wavelength of 450 nm (reference, 650 nm).

### EdU assay

For EdU assay, cells were pre-cultured with EdU for 2 h using a Mixture Reagent Kit (C10338-3, RiboBio) following the manufacturer's protocol. The cells were collected in a 15 mL centrifuge tube, then fixed with 4% paraformaldehyde for 30 min, and incubated with 0.5% Triton-X-100 in PBS for 20 min. After incubation with Apollo staining solution for 10 min, the cells were immediately detected by flow cytometry.

### Apoptosis assay

Cells undergoing apoptosis were assessed using a FITC Annexin V Apoptosis Detection Kit I (BD Biosciences). Cells were carefully dissociated into a single-cell suspension using Accutase solution, washed twice with ice-cold PBS, and resuspended in 100 µL of 1×binding buffer at a density of 1×10^6^ cells/mL. Subsequently, Annexin-V (5 µL) and PI (5 µL) solution, which label early and late apoptotic cells, respectively, were added to the cells. The mixture was then vortexed gently and incubated for 15 min at room temperature in the dark. After staining, 400 µL of 1× binding buffer was added, and the cells were analyzed using a FACSCanto II (Becton Dickinson).

### Microarray analysis

Total RNA was extracted from the two independent samples (siControl versus si*ESRG*) using the RNAiso reagent (Takara). Following the manufacturer's instructions, RNA for analysis was labeled and hybridized onto the Illumina Human HT-12 v4 array by GenomeScan.

### PCR array analysis of the human p53 signaling pathway

Gene expression profiles related to the p53 signaling pathway were analyzed using a Human p53 signaling pathway RT Profiler PCR Array (SABiosciences) according to the manufacturer's instructions (QIAGEN). Total RNA was isolated using the RNeasy Mini Kit (QIAGEN) and purified with the RNase-Free DNase Set (QIAGEN), and cDNAs were synthesized using the RT First Strand Kit (QIAGEN) on a thermocycler PCR machine (Bio-Rad). Subsequently, RT SYBR Green Mastermix (QIAGEN) and the RT Profiler PCR Array Human p53 Signaling Pathway (PAHS-027Z, QIAGEN) were used to evaluate the expression profiles of 84 key genes involved in apoptosis, cell cycle, cell growth, proliferation, differentiation, and DNA repair on a CFX96 qPCR machine (Bio-Rad). Data analysis was performed using PCR Array Data Analysis web portal (www.SABiosciences.com/pcrarraydataanalysis.php). Gene expression changes with a fold change greater than 2 were considered biologically significant.

### Immunofluorescence-FISH (IF-FISH)

The cells were fixed with 4% paraformaldehyde, digested with protease K and incubated at 37 °C for 1 h. Then, a FISH probe was added, and the mixture was incubated at 4 °C overnight followed by nuclear staining the next day. A FISH/IF assay is a combination of RNA FISH and protein IF as described previously 32, 33.

### Northern Blot analysis

An *ESRG*-specific DNA probe (*ESRG*-F: 5' ATGAAAGGGAAGACATACAA 3', *ESRG*-R: 5' TGAACATAGCAAGGGAAA 3') with a length of 314 bp was obtained by PCR. Total RNA was extracted from cell lines by TRIzol method and detected by northern blotting. The RNA samples were subjected to agarose gel electrophoresis under denaturation conditions, and then the RNA on the gel was transferred to the solid phase support *in situ*. After dry roasting, the samples were hybridized with an *ESRG* probe and detected by specific autoradiography.

### RNA pulldown and mass spectrometry analysis

*In vitro* biotin-labeled RNAs (*ESRG*, its antisense RNA, and four truncated RNAs from *ESRG*) were transcribed with a biotin RNA labeling mix (Roche) and T7 RNA polymerase (Roche), and then purified with an RNeasy Mini Kit (QIAGEN) after treatment with RNase-free DNase I (Promega). Three micrograms of biotinylated RNA was heated to 95 °C for 2 min, and then left at room temperature (RT) for 20 min to form the proper secondary structure before use. Biotinylated RNA was incubated with cellular protein extracts and streptavidin agarose beads (Invitrogen), and then the pulled down proteins were separated on SDS‒PAGE gels followed by mass spectrometry.

### MMP (mitochondrial membrane potential) analysis

MMP was analyzed by fluorescence microscopy using the mitochondrial membrane potential assay kit with JC-1 (Beyotime) following the manufacturer's instructions. Briefly, cells were rinsed with PBS, incubated with a mixture of 1 mL of culture medium and 1 mL of JC-1 working solution for 20 min at 37°C, washed twice with JC-1 buffer solution, and analyzed using a Nikon Eclipse TE2000-S inverted microscope (Nikon). At high MMPs, JC-1 can form aggregates in the mitochondrial matrix and fluoresces red, whereas at low MMP, JC-1 exists in the monomeric form outside the mitochondrial matrix and fluoresces green fluorescence. The fluorescence intensity shift from red (JC-1 aggregates) to green (JC-1 monomers) indicates a decrease in the MMP and early stages of apoptosis.

### RIP (RNA immunoprecipitation)

The RIP assay was performed with the EZ-Magna RIP Kit (Millipore, USA) according to the manufacturer's instructions. Anti-MCM2 antibody was purchased from Proteintech. The coprecipitated RNAs associated with MCM2 were extracted with the TRIzol reagent, and *ESRG* enrichment was examined using qPCR. Enrichment associated with normal rabbit IgG served as the controls.

### Co-IP (Co-immunoprecipitation)

For the co-immunoprecipitation (Co-IP) assay, proteins were harvested in lysis buffer (Beyotime Biotechnology) and supplemented with protease inhibitor (Selleck). After culturing with primary antibody as indicated in the figure legends, or mouse IgG for 4 h, protein A/G PLUS beads (sc-2003, Santa Cruz Biotechnology) were added and incubated overnight. The precipitants were washed at least three times with lysis buffer.

### Luciferase reporter construction and luciferase activity assay

DNA fragments (approximately 2000 bp) upstream of the transcription starting site of the *ESRG* gene were PCR-amplified using genomic DNA from hESCs as a template. The primers used were 5'-CGGGGTACCCCACCAAAACTTACT-3' (forwards, -2049 to -2029; the underlined part indicates a *Kpn*I restriction site) and 5'-TCCCCCGGGAGAGAGTCACGAAGGGAGATAA-3' (reverse, -12 to +11; the underlined part indicates a *Sma*I restriction site). PCR fragments were digested by *Kpn* I and *Sma*I, inserted into a pGL3-basic vector and sequenced. To construct the p*ESRG*-OCT4B-luciferase reporter, DNA fragments encompassing OCT4-binding elements upstream of the *ESRG* transcription start sites were PCR-amplified using hESC genomic DNA. The primers used were as follows: wild-type, 5'-CGGGGTACCGGAT*ATTTGCAT*TCGCTAGAGAAT-3' (forwards primer, underlined part indicates a *Kpn*I restriction site, italicized bases represent the OCT4-binding site [-1713 to -1721]) and 5'-TCCCCCGGGAAAGCACGAGGGGTA-3' (reverse primer, underlined part indicates a *Sma*I restriction site). PCR fragments were digested by *Kpn* I and *Sma* I, inserted into a pGL3-promoter vector, and sequenced. The same procedure was followed to obtain the reporter constructs p*ESRG*-*OCT4*B-luciferase with mutations in the OCT4-binding elements of the *ESRG* promoter regions; however, the following was used as the forwards primer: (5'-CGGGGTACCGGATTTAGCGTTCGCTAGAGAA T-3', in which the dashed line indicates the element substitutions).

hPSCs were plated in a 6-well plate at a density of 2×10^5^ cells/well. After 24 h, pGL3 reporter plasmids were introduced into hESCs using FuGENE HD transfection reagent. Forty-eight hours later, the cells were washed twice and suspended in 500 μL of reporter lysis buffer (Promega), and firefly luciferase activity was measured using the Dual-Luciferase reporter assay system (Promega) and a GloMax 20/20 luminometer (Promega) according to the manufacturer's protocol. The Renilla luciferase plasmid pRL-TK (Promega) was cotransfected as an internal control. The pGL3 control plasmid was used as a positive control. siOCT4 was transfected into hESCs with Lipofectamine RNAiMAX using the protocol described for RNAi. The corresponding target mRNA sequence for the siOCT4 was GCTTCAAGAACATGTGTAA. Furthermore, the ORF of OCT4 was cloned from cDNA from H9 hESCs and inserted into the *Bam* HI/*Xba* I restriction site of the pcDNA3.1 vector to obtain the OCT4 expression vector (pcDNA3.1-*OCT4*), which was then transfected into 293T cells using FuGENE HD according to the manufacturer's protocol.

### ChIP assay

ChIP analysis was performed according to the instructions provided in the EZ-ChIP Kit (Millipore). Chromatin was incubated overnight with anti-OCT4 antibody (Santa Cruz) or control rabbit IgG (Millipore). Precipitated DNA was further purified and amplified with PCR primers (Forwards: 5'-TGGCATAGCACTGAAAGG-3'; Reverse: 5'-CTGACTGGCAGTTGGTTG-3') surrounding the OCT4 binding site upstream of the *ESRG* gene.

### Mice and teratoma assay

NOD-PrkdcscidIL2rgem2/SMOC (NSG) mice (male, 4-6 weeks) were purchased from the Shanghai Model Organisms. Then, the mice were bred in specific pathogen free (SPF) conditions, and the mouse experiments were approved by the ethics committee of the Central South University. According to the methods of a previous study 34, H9-TetR-sh*ESRG* cells were pretreated with Y27632 for 3 h prior to injection, and mice received Dox in their drinking water 1 week before injection and during the whole period of teratoma formation (H9-TetR-sh*ESRG*+Dox). A 200 µL cell suspension mixture containing 50% Matrigel and 1×10^7^ Y27632-treated cells was injected into the hindlimb muscles of the NSG mice. The teratoma tissues were removed 6-8 weeks after injection and fixed with 10% neutral formalin buffer. Then, tissue sections were prepared and stained with hematoxylin and eosin.

### Comet assay

H9 hESCs were transfected with si*ESRG* and siControl for 48 h and then harvested in PBS. The comet assay was performed according to manufacturer's protocol (KGA240, KeyGEN BioTECH). In detail, three layers of gels were prepared on slides with agarose at different dissolution points, in which the cell suspension was mixed with low melting agarose (LMA) as the second layer of gel. The slides were lysed by placing them in a dish with 20 mL of lysis buffer and then placed in the electrophoretic solution for unwinding. Finally, the samples were stained with PI and observed under a fluorescence microscope for capture image.

### Statistical analyses

All analyses were performed using SPSS Statistics 19.0 software. The error bars represent the standard deviation (SD) of three independent experiments, and statistically significant differences by Student's *t* test are indicated by *, **, and ***, (or #, ##, and ###) indicating *P* < 0.05, *P* < 0.01, and *P* < 0.001, respectively. *P* values < 0.05 were considered significant.

## Results

### *ESRG* maintains pluripotency of hPSCs

**Figure 1:**
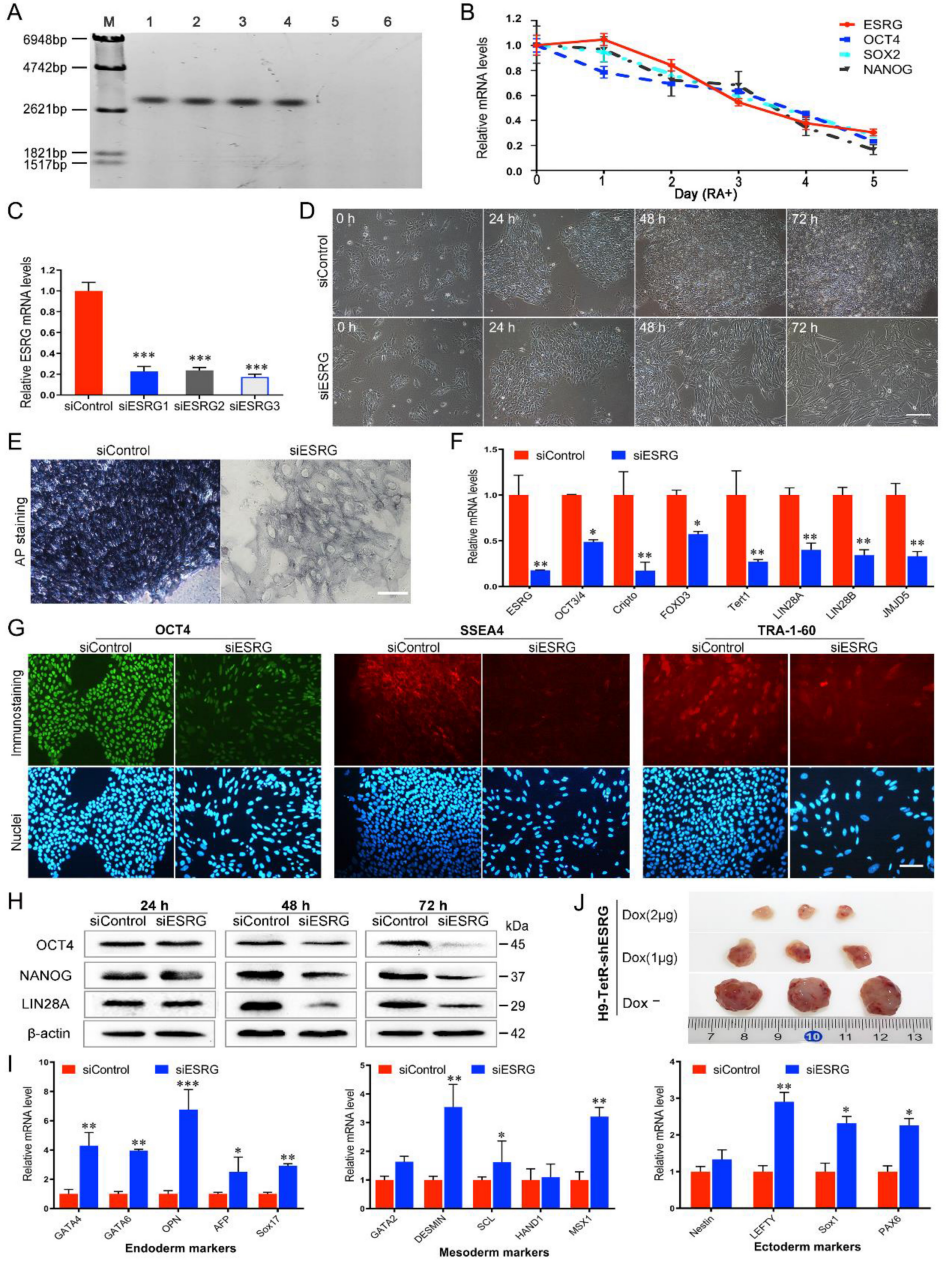
** ESRG maintains pluripotency of hPSCs. (A)** Northern blot analysis of *ESRG* with DIG Probe in various cell lines. Lanes 1 to 6 were H9, H1, RC1-iPSC, RC2-iPSC, HFF and 293T cell lines, respectively. **(B)** After the differentiation of H9 hESCs was induced by retinoic acid (RA) *in vitro*, the expression of *ESRG*, OCT4, SOX2 and NANOG was detected by qPCR. **(C)** The RNA level of *ESRG* was analyzed by qPCR after transfecting each of the three effective siRNA sequences (si*ESRG*1, si*ESRG*2, si*ESRG*3) for 48 h. **(D)** Brightfield images of H9 hESCs transfected with si*ESRG* (herein si*ESRG*1, the same below). Scale bar, 100 μm. **(E)** AP staining was performed in H9 hESCs transfected with si*ESRG* or siControl, and the dark blue color indicated undifferentiated, AP-positive cells. Scale bar, 100 μm.** (F)** The expression of pluripotency marker genes was analyzed by qPCR in H9 hESCs transfected with si*ESRG* or siControl.** (G)** H9 hESCs transfected with si*ESRG* or siControl were stained with anti-OCT4, anti-SSEA4 and anti-TRA-1-60 antibodies (upper panel). Hoechst 33342 was used to label cell nuclei (lower panel). Scale bar, 100 μm.** (H)** Protein levels of OCT4, NANOG and LIN28A were detected by Western blot analysis in *ESRG* knockdown and control H9 hESCs.** (I)** The expression of endoderm, mesoderm, ectoderm and trophectoderm marker genes was analyzed by qPCR in H9 hESCs transfected with si*ESRG*. **(J)** Teratomas were derived from H9-TetR-sh*ESRG* cells inoculated NSG mice with or without treatment of Dox. Data are presented as mean ± SD. *P < 0.05, **P < 0.01, ***P < 0.001 (two-tailed Student's *t* test).

*ESRG* is located on human chromosome 3p14.3 and consists of four exons and three introns with a full length of 3153 nt 13. Its high and exclusive expression in hPSCs was verified by northern blotting, which showed high expression of *ESRG* in hPSC lines such as H9, H1, RC1 and RC2-iPSCs but not in HFF and 293T cells (Fig. 1A). To demonstrate the dynamic changes in *ESRG* expression during hPSC differentiation, we induced hESC differentiation *in vitro* with retinoic acid (RA). As shown in Fig. 1B, along with the differentiation of H9 hESCs, the expression of *ESRG* dramatically decreased to undetectable levels, and the change trend of its expression level was similar to those of *OCT4*, *SOX2* and *NANOG*. By *in situ* hybridization (ISH), we found that *ESRG* was mainly located in the nucleus of hPSCs, with a small part in the cytoplasm (Fig. S1A), which was further confirmed by cellular fractionation assays (Fig. S1B). Thus, *ESRG* may primarily exert its biological function in the nucleus.

To assess the functional significance of *ESRG* in hPSCs, twenty-five sets of siRNAs targeting different regions of the *ESRG* sequence were designed and transfected into H9 hESCs. Cy3-labeled negative control siRNA (5'-Cy3-siControl) was used to evaluate the transfection efficiency. As shown in Fig. S1C, nearly 100% of the cells were Cy3 positive, indicating that siRNA could be effectively delivered into hPSCs. Then, three siRNA sequences (si2364, si2243, and si2621) with the highest knockdown efficiency matching the *ESRG* sequence starting at nucleotides 2364, 2243 and 2621, respectively (Fig. S1D-F), were selected, renamed as si*ESRG*1, si*ESRG*2 and si*ESRG*3, and used in the subsequent experiments. The qPCR results showed that the expression level of *ESRG* decreased by more than 75% at 48 h after transfection with any of the three sets of siRNAs (Fig. 1C). In addition, a panel of sh*ESRG* lentiviruses was also used to silence *ESRG* gene expression (with an interference efficiency >70%) in hPSCs (Fig. S1G).

Then, we knocked down *ESRG* expression in hPSCs (herein si*ESRG*1, the same below). Forty-eight hours after the transfection, the hPSCs with reduced *ESRG* expression appeared dispersed and slender, and did not form compact colonies, whereas the cells transfected with siControl retained the typical cell and colony morphology of undifferentiated hPSCs (Fig. 1D and Fig. S1I), and these differences were even more obvious after 72 h (Fig. 1D). More importantly, targeting *ESRG* with three effective siRNA sequences caused identical phenotypic changes in hPSCs (Fig. 1D and Fig. S1J-K), confirming that this phenotype was not a result of off-target effects of the siRNAs. Furthermore, specific knockdown of *ESRG* in hPSCs by lentivirus carrying sh*ESRG* caused phenotypic changes similar to those caused by the highly efficient transfection of si*ESRG* (Fig. S1H).

Differentiation was marked by the loss of alkaline phosphatase (AP) activity. AP staining analysis showed an almost complete loss of AP activity in the si*ESRG* group compared with the apparent AP activity in the siControl group (Fig. 1E and Fig. S2A), indicating that knockdown of *ESRG* caused the differentiation of hPSCs. In addition, we observed that knockdown of *ESRG* in hPSCs resulted in significant downregulation of pluripotency markers such as *OCT3/4*, *Cripto*, *FOXD3*, *Tert1*, *LIN28* and *JMJD5* (Fig. 1F and Fig. S2B). Subsequently, we examined the protein expression levels of OCT4 and NANOG. As shown in Fig. 1G, the fluorescence signals of OCT4, SSEA4 and TRA-1-60 in the cells transfected with si*ESRG* were weaker than those in the control cells. As expected, knockdown of *ESRG* caused significant downregulation of the steady-state protein levels of OCT4 and NANOG compared with control hPSCs, as detected by western blot (Fig. 1H and Fig. S2C). The altered differentiation state of the hPSCs after knockdown of *ESRG* in H9 was further identified by qPCR based on the expression of triploblastic marker genes, including endoderm-specific, mesoderm-specific and ectoderm-specific markers. As shown in Fig. 1I and Fig. S2D, these germ layer markers were all significantly upregulated, with the endoderm-specific markers increasing the most, suggesting that *ESRG* plays a role in repressing hPSC differentiation.

Furthermore, by transfecting pCAG-TetRnls and pSUPERIOR-sh*OCT4*/-sh*ESRG* vectors into H9 hESCs (Fig. S2E-F), we established doxycycline (Dox)-inducible *OCT4*/*ESRG* RNAi systems (H9-TetR-sh*OCT4*/H9-TetR-sh*ESRG* cells) allowing knockdown of OCT4 (Fig. S2G-H) or *ESRG* (Fig. S2I-J) selectively in hPSCs. Inducible knockdown of *ESRG* in hESCs resulted in obvious phenotypic changes and cell differentiation, as expected. To examine the role of *ESRG* in modulating the differentiation of hPSCs *in vivo*, a teratoma formation assay was performed, and H9-TetR-sh*ESRG* cells with or without Dox were subcutaneously injected into NOD-*Prkdc*^scid^*IL2rg*^em2^/SMOC (NSG) mice. After approximately 6-8 weeks, the teratomas formed by injection of H9-TetR-sh*ESRG* cells with Dox were postponed and much smaller than those formed in the control group without Dox, and the higher the Dox concentration was, the smaller the tumors formed (Fig. 1J and Fig. S2K). When the H9-TetR-sh*ESRG* cells were pretreated with Dox for 5 days prior to injection, no teratoma developed (data not shown). Together, these* in vitro* and *in vivo* results suggest that *ESRG* functionally maintains pluripotency of hPSCs.

### *ESRG* is essential for hPSC self-renewal

**Figure 2:**
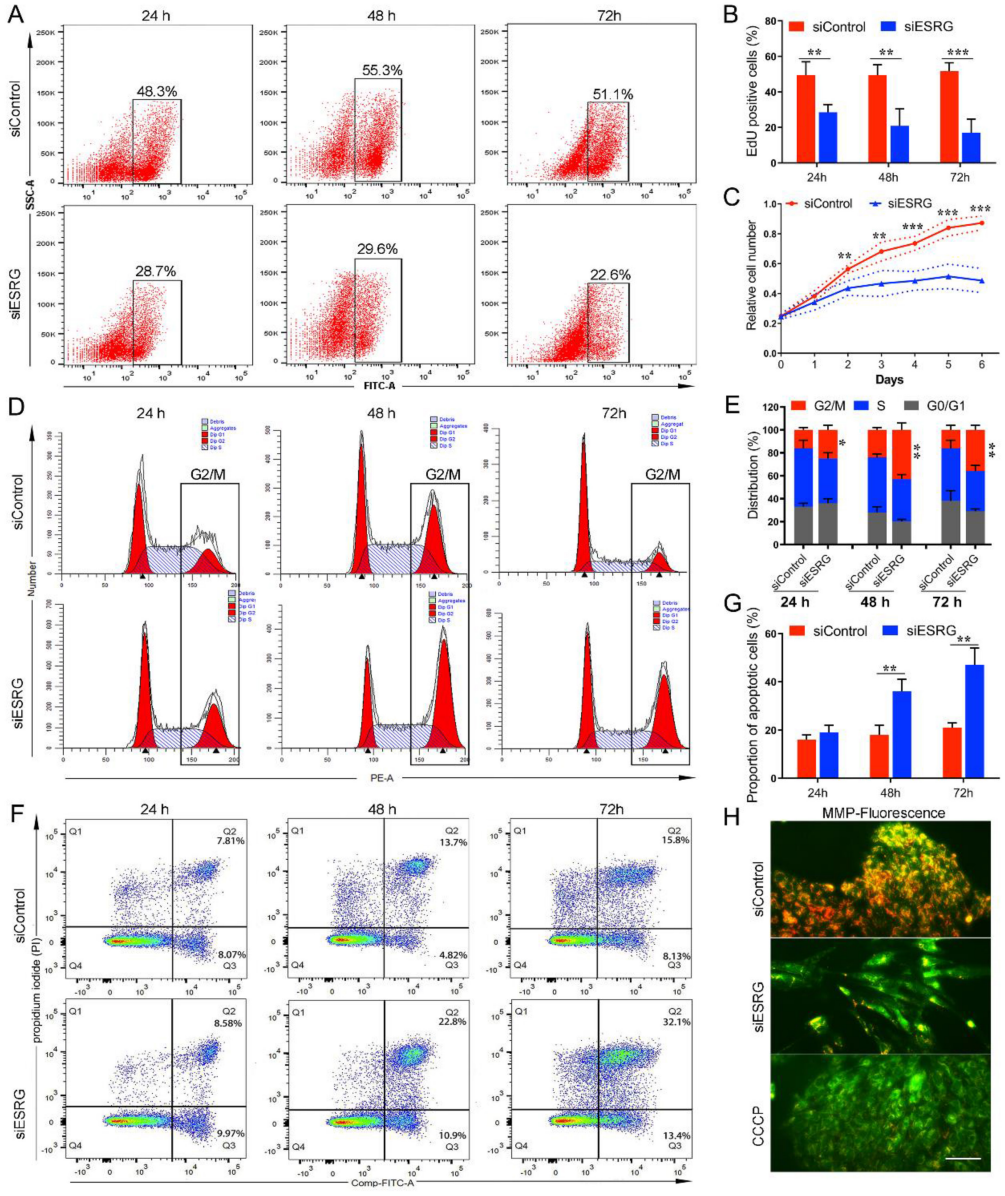
***ESRG* is essential for hPSC self-renewal. (A)** EdU assay was analyzed by flow cytometry in H9 hESCs transfected with si*ESRG* or siControl at 24 h (first column), 48 h (second column) and 72 h (third column) after transfection.** (B)** The quantified analysis of EdU positive cells.** (C)** Data from the CCK-8 assay of *ESRG* knockdown and control H9 hESCs. **(D)** Cell cycle distribution was analyzed by flow cytometry in H9 hESCs transfected with si*ESRG* or siControl at 24 h (first column), 48 h (second column) and 72 h (third column) after transfection.** (E)** The quantified analysis of cell cycle distribution.** (F)** Apoptosis was analyzed using a FITC/Annexin V apoptosis assay in H9 hESCs transfected with si*ESRG* or siControl at 24 h (left column), 48 h (middle column) and 72 h (right column) after transfection. **(G)** The quantified analysis of the apoptosis assay. **(H)** H9 hESCs transfected with si*ESRG* or siControl were stained with JC-1 to evaluate the MMP. Scale bar, 100 μm. All representative examples of the data from at least three independent experiments are shown. Data are presented as mean ± SD. *P < 0.05, **P < 0.01, ***P < 0.001 (two-tailed Student's *t* test).

In addition to the differentiation induced by knockdown of *ESRG*, cell proliferation was decreased, suggesting that *ESRG* plays a critical role in maintaining hPSC proliferation and self-renewal. To investigate the effects of *ESRG* knockdown on hPSC proliferation and survival, we employed an EdU (5-ethynyl-2'-deoxyuridine) incorporation assay to quantitatively assess cell proliferation. As expected, *ESRG* knockdown cells showed less EdU incorporation than control cells (Fig. 2A-B and Fig. S3A), which was further confirmed via CCK-8 assay, and the cell growth rate of *ESRG* knockdown hPSCs decreased significantly compared with the control cells (Fig. 2C).

To further characterize the role of *ESRG* in hPSC survival, cell cycle analysis was performed using PI staining. The percentage of *ESRG* knockdown cells in the G2/M phase was significantly higher than that of the controls, showing that hPSCs subjected to *ESRG* silencing accumulated in G2/M phase of the cell cycle, with a concomitant decrease in the fraction of cells in G0/G1 or S phases (Fig. 2D-E and Fig. S3B). In addition, EdU/PI staining was used to analyze cell cycle distribution, and the results were consistent with those of PI staining alone (Fig. S3D).

Moreover, we found that the number of floating dead cells reminiscent of apoptotic cells in *ESRG*-silenced hPSC cultures was increased compared with that in cultures of control cells. Therefore, changes in apoptosis were detected using a FITC Annexin V apoptosis assay. The Annexin V/PI apoptotic cell population in *ESRG*-silenced cells was significantly increased at 48 h and 72 h after transfection (Fig. 2F-G and Fig. S3C). Next, analysis of the mitochondrial membrane potential (MMP) showed that *ESRG* knockdown cells exhibited a remarkable decrease in MMP compared with control cells (Fig. 2H), suggesting that *ESRG* knockdown resulted in hPSC apoptosis. Together, these results indicated the essential role of *ESRG* in sustaining hPSC cell survival and self-renewal. Similar results were obtained after the transfection with the three *ESRG* siRNAs (Fig. 2 and Fig. S3E-J).

To further confirm that these phenotypes and functions caused by *ESRG* knockdown are not the result of RNAi off-targets effects, we constructed an *ESRG* adenovirus vector and performed a rescue analysis. The results showed that overexpression of *ESRG* rescued the morphological changes caused by *ESRG* knockdown and reversed the loss of pluripotency and the inhibition of cell proliferation (Fig. S4). These results confirmed that this series of biological effects are indeed caused by *ESRG* knockdown, indicating that *ESRG* plays an important role in the maintenance of the self-renewal and pluripotency of hPSCs.

### *ESRG* is transcriptionally regulated by OCT4

**Figure 3:**
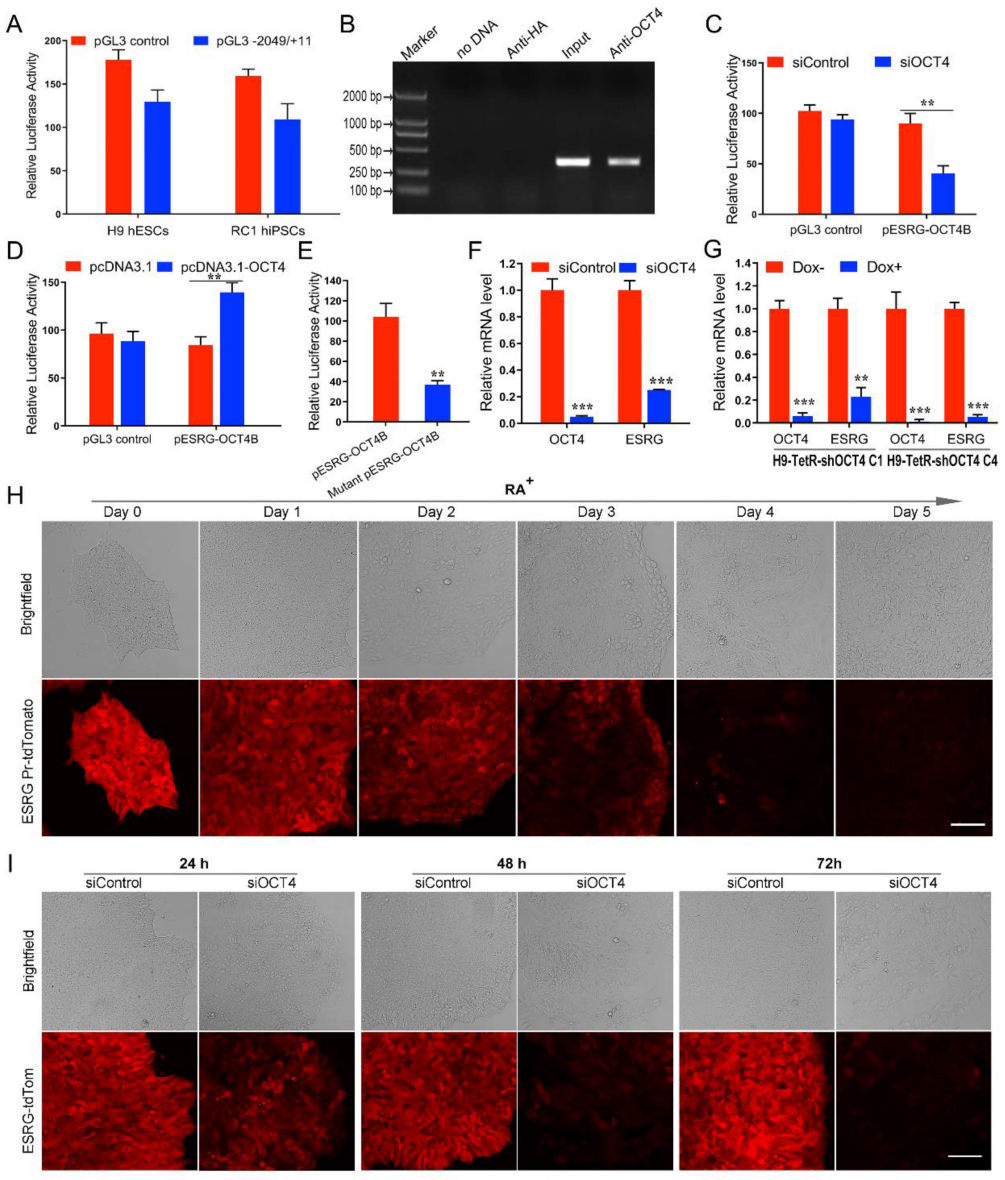
***ESRG* is transcriptionally regulated by OCT4. (A)** The relative luciferase activities in hPSCs (H9 and RC1) transfected with a luciferase vector containing the -2049/+11 fragment of* ESRG* are similar to that of the pGL3 control vector.** (B)** A ChIP assay was performed in H9 hESCs lysates. **(C)** The relative luciferase activity in H9 hESCs co-transfected with either siOCT4 or siControl and either a p*ESRG*-*OCT4*B-luciferase reporter plasmid or a pGL3 control plasmid. **(D)** The relative luciferase activity in 293T cells co-transfected with either pcDNA3.1-*OCT4* or pcDNA3.1 (negative control) and either p*ESRG*-OCT4B-luciferase reporter plasmid or pGL3 control plasmid. **(E)** Detection of luciferase activity in H9 hESCs transfected with a mutant p*ESRG*-*OCT4*B-luciferase reporter plasmid harboring a mutated OCT4 binding site and a wild-type p*ESRG*-*OCT4*B-luciferase reporter plasmid. The relative luciferase activity represents the ratio of firefly luciferase activity to Renilla luciferase activity (internal control). **(F)** QPCR analysis was performed for *OCT4* and *ESRG* expression in H9 hESCs transfected with siOCT4 or siControl. **(G)** QPCR analysis of *OCT4* and *ESRG* expression in two Dox inducible H9-TetR-sh*OCT4* cell clones (C1 and C4) treated with or without Dox. **(H)** Fluorescence intensity of *ESRG*-tdTom cells transfected with siOCT4 at 24 h, 48 h and 72 h. Red fluorescence represents *ESRG* expression. Scale bar, 100 μm. **(I)** Phase images of *ESRG* Pr-tdTomato reporter H9 hESCs treated with RA for 5 days. Red fluorescence represents *ESRG* expression. Scale bar, 100 μm. Data are presented as mean ± SD. **P < 0.01, and ***P < 0.001 by two-tailed Student's *t* test.

The core promoter region of *ESRG* was predicted to be -1,346 to -1,297 bp upstream of the transcription start site 13. To identify the activity of the predicted promoter region, we amplified a fragment spanning from position -2049 bp to +11 bp (-2049/+11) of *ESRG*, cloned it into a pGL3 basic vector (pGL3-2049/+11), and transfected the vector into hPSCs. The results from the luciferase reporter assay showed that this fragment exhibited strong promoter activity compared with a blank control, which suggested that the -2049/+11 fragment plays an important role in triggering the transcription of *ESRG* in hPSCs (Fig. 3A).

Next, we used the online tool TESS (Neural Network Promoter Prediction) (http://fruitfly.org/seq_tools/promoter.html) to analyze the transcription factors that might regulate *ESRG* expression and found that the OCT4 binding site (ATTTGCAT) was -1714 to -1707 bp upstream of *ESRG*. Chromatin immunoprecipitation (ChIP) experiments verified that OCT4 directly bound to the OCT4 binding site within the *ESRG* promoter (Fig. 3B). To explore the possible role of OCT4 in regulating *ESRG* transcription, siRNA targeting *OCT4* mRNA was cotransfected into hPSCs with a luciferase reporter vector containing the *ESRG* promoter that includes the OCT4 binding site (p*ESRG*-*OCT4*B-luciferase). Downregulating OCT4 decreased the luciferase activity of the reporter vector group as compared with the control (Fig. 3C). Furthermore, pcDNA 3.1-*OCT4*, the plasmid expressing OCT4, was cotransfected with the p*ESRG*-*OCT4*B-luciferase plasmid into 293T cells without endogenous OCT4 expression. A luciferase activity assay showed that ectopic *OCT4* expression significantly increased the luciferase activity of the reporter vector group compared with that of negative control (Fig. 3D). These results suggested that OCT4 positively regulates *ESRG* transcription.

To further confirm that OCT4 regulates the activity of the *ESRG* promoter, we constructed a mutated luciferase reporter vector containing a mutated OCT4 binding consensus sequence with two nucleotides replaced (mutant p*ESRG*-*OCT4*B-luciferase). The wild-type and mutant reporter vectors were transfected into hPSCs respectively, and the luciferase activity was analyzed at 48 h after transfection. As shown in Fig. 3E, the luciferase activity in the mutant reporter vector group was significantly lower than that in the wild-type reporter vector group, suggesting that the predicted OCT4 binding site upstream of *ESRG* acts as a positive cis-element in regulating *ESRG* transcription. qPCR analysis also indicated that silencing OCT4 downregulated *ESRG* in both siOCT4-transfected cells (Fig. 3F) and Tet-inducible sh*OCT4* hPSCs (H9-TetR-sh*OCT4*) (Fig. 3G). Moreover, the level of *ESRG* downregulation was positively correlated with the silencing efficiency of OCT4, indicating that *ESRG* is a downstream target of OCT4 (Fig. 3F-G).

In our previous study, we successfully established an *ESRG* Pr-tdTomato reporter H9 cell line by homologous recombination technology to monitor the expression of *ESRG* for further study of its function 28. We found that the fluorescence intensity decreased gradually when *ESRG* Pr-tdTomato reporter H9 cells were treated with RA (Fig. 3H), indicating that the expression level of *ESRG* decreased gradually with cell differentiation. Furthermore, after the expression of OCT4 was knocked down, the red fluorescence reflecting the activity of the *ESRG* promoter decreased gradually over time (Fig. 3I). All of these findings further demonstrated that OCT4 positively and directly regulates *ESRG* expression in hPSCs.

### *ESRG* functions in hPSCs by directly binding MCM2

We assessed the binding proteins of *ESRG* using RNA pull-down assays and mass spectrometry analysis (Fig. S5A). Using the antisense sequence of *ESRG* as a negative control, we obtained seventy-two proteins specifically binding to *ESRG*. Among them, we found that the DNA replication licensing factor MCM2 was specifically associated with *ESRG* (Table S1). The expression level of MCMs is positively correlated with cell proliferation and regeneration 35, 36. As a component of the MCM2-7 complex, MCM2 is a putative replicative helicase that is essential for the initiation and elongation of “once per cell cycle” DNA replication in eukaryotic cells 37. As we demonstrated in this study, knockdown of *ESRG* resulted in a serious impact on DNA replication and cell proliferation of hPSCs. Therefore, we hypothesized that *ESRG* might function in hPSCs by interacting with MCM2.

**Figure 4:**
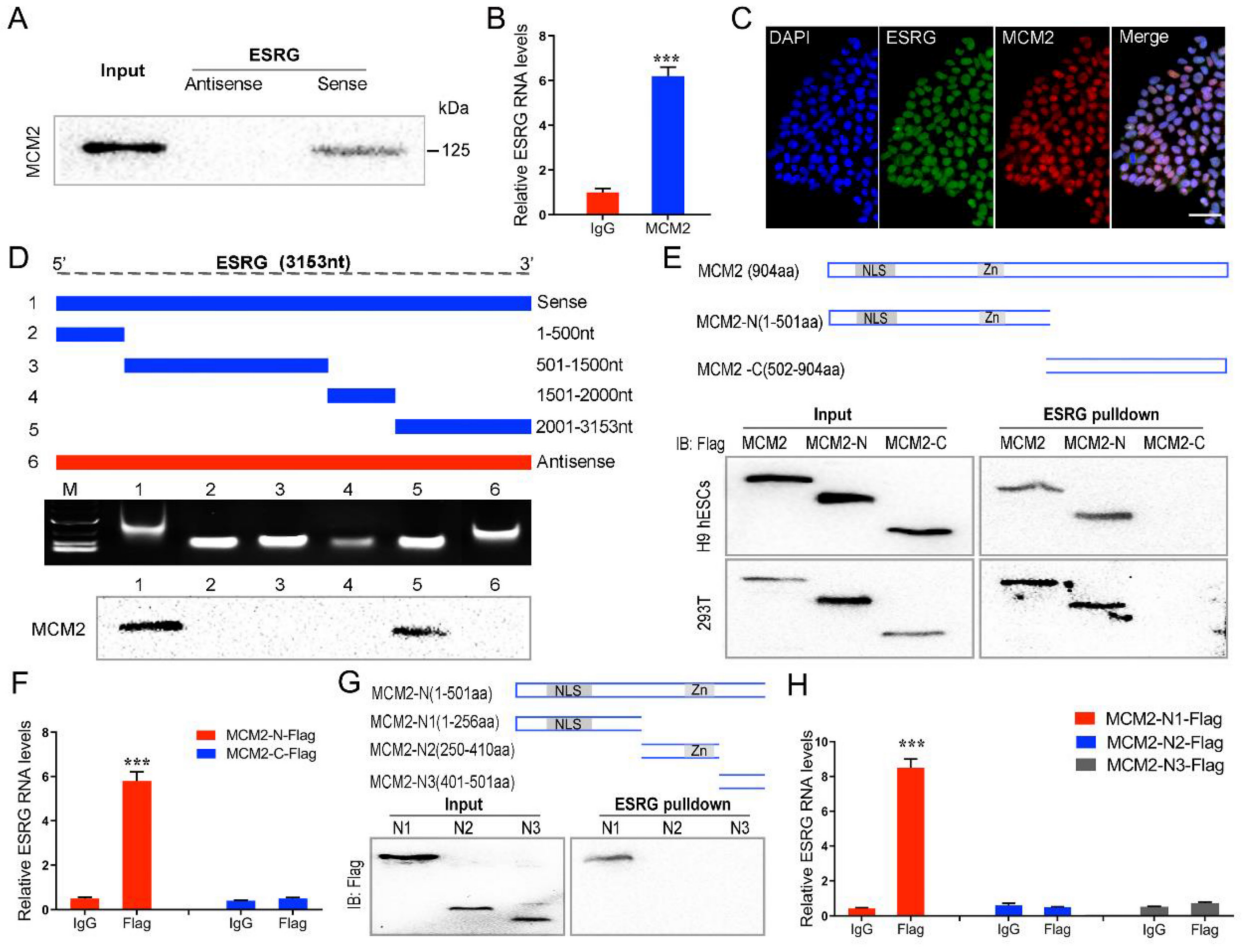
***ESRG* functions in hPSCs by directly binding MCM2. (A)** RNA pulldown showed binding between *ESRG* and MCM2 in H9 hESCs. **(B)** The interaction of *ESRG* and MCM2 was detected through RIP assay in H9 hESCs. **(C)** *ESRG* was visualized by RNA-FISH, and MCM2 was stained by immunofluorescence in H9 hESCs. Scale bar, 100 μm.** (D)** Deletion mapping of the MCM2-binding domain in *ESRG*. Top, diagrams of full-length *ESRG* and the deletion fragments. Middle, the *in vitro*-transcribed full-length *ESRG* and deletion fragments with the correct sizes are indicated. Bottom, immunoblot analysis for MCM2 in the protein samples pulled down by different *ESRG* constructs. **(E)** The immunoblot analysis of Flag-tagged MCM2 [wild-type vs. domain truncation mutants (MCM2-N and MCM2-C)] retrieved by *in vitro*-transcribed biotinylated *ESRG*. The domain structure of MCM2 is shown above. **(F)** The isolated MCM2-N domain is sufficient for binding to *ESRG*, as demonstrated using the RIP assay. **(G)** The immunoblot analysis of Flag-tagged MCM2 [domain truncation mutants (MCM2-N1, MCM2-N2 and MCM2-N3)] retrieved by *in vitro*-transcribed biotinylated *ESRG*. The domain structure of MCM2-N is shown above. **(H)** The isolated MCM2-N1 domain is sufficient for binding to *ESRG*, as demonstrated using the RIP assay. Data are presented as mean ± SD. ***P < 0.001 by two-tailed Student's *t* test.

To prove this hypothesis, we first verified the association of *ESRG* with MCM2 through RNA pull-down. MCM2 was enriched in the sense strand groups compared with the negative control antisense groups in hPSCs (Fig. 4A and Fig. S5B). RNA immunoprecipitation (RIP) assays also confirmed an enrichment of *ESRG* in the complexes precipitated with the antibody against MCM2 (Fig. 4B and Fig. S5C). In addition, immunofluorescence combined with RNA-FISH assays demonstrated that *ESRG* and MCM2 were mainly colocalized in the nuclei of hPSCs, further confirming their binding relationship (Fig. 4C and Fig. S5D).

Next, a series of *ESRG* deletion mutants were constructed to determine the nucleotides in *ESRG* that are responsible for binding to MCM2. As shown in Fig. 4D, further mapping of the region with RNA pull-down indicated that the 2,001-3,153 nt domain was required for the *ESRG*-MCM2 interaction in hPSCs. In the same way, to map the region or regions within MCM2 that interact with *ESRG*, we tested the binding of MCM2 truncation variants to *ESRG*. First, we constructed two MCM2 domains, namely, MCM2-N and MCM2-C (Fig. 4E). After pull-down assays and RIP experiments in H9 hESCs and 293T cells, we found that MCM2-N, but not the MCM2-C domain of MCM2, was required for *ESRG* binding (Fig. 4E -F). Thus, the MCM2-N domain of MCM2 was necessary and sufficient for *ESRG* binding. We further decomposed MCM2-N into MCM2-N1, MCM2-N2 and MCM2-N3 domains and found that *ESRG* specifically binds to the MCM2-N1 domain (Fig. 4G-H). Together, these data revealed that residues 1-256 of MCM2 and residues 2,001-3,153 of *ESRG* are critical for the *ESRG*-MCM2 interaction.

### *ESRG* sustains the steady-state level and nuclear location of MCM2

**Figure 5:**
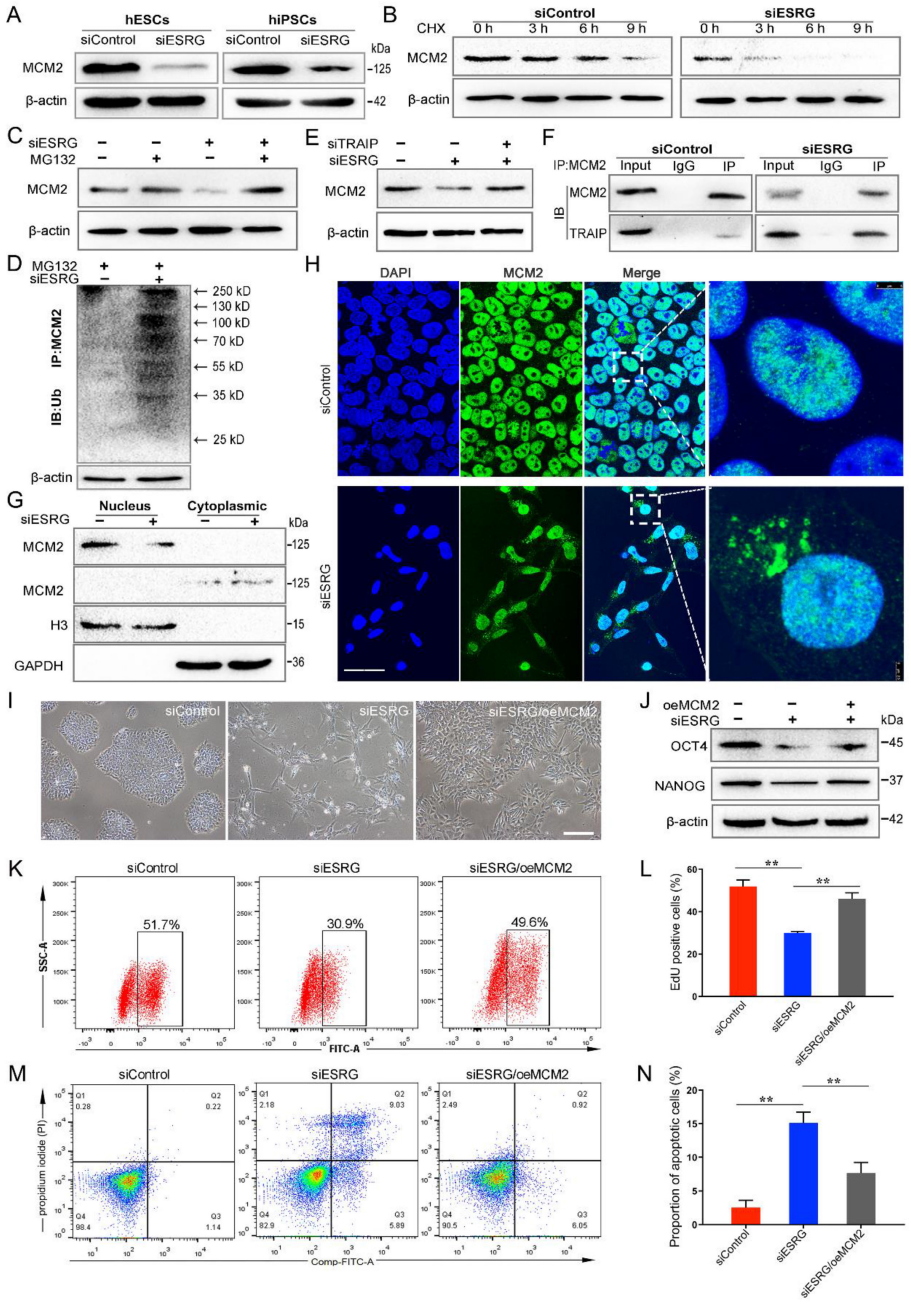
***ESRG* sustains the steady-state levels and nuclear location of MCM2.** (**A**) The expression of MCM2 was detected by Western blot after transfection of H9 cells and RC1-iPSCs with si*ESRG*. (**B**) The expression of MCM2 in H9 cells (siControl vs si*ESRG*) was detected by Western blot after treatment with CHX (20 μg/mL) for various time periods respectively. (**C**) H9 cells were transfected with *ESRG* siRNA and pre-incubated with MG-132 (20 μM) for 4 h. Cell lysate was immunoblotted by anti-MCM2. (**D**) H9 cells were pre-incubated with MG-132 (20 μM) for 4h. Ub was immunoprecipitated (IP) by anti-MCM2 and immunoblotted (IB) by anti-Ub. The ubiquitination of MCM2 protein was detected after *ESRG* knockdown. (**E**) H9 cells were treated with TRAIP siRNA followed by *ESRG* knockdown. MCM2 protein expression was detected by Western blot. (**F**) The interaction of MCM2 and TRAIP was measured by Co-IP after treated with siControl or si*ESRG* in H9 hESCs. (**G**) The nuclear and cytoplasmic extracts from H9 cells after *ESRG* knockdown were detected by Western blot. (**H**) MCM2 was visualized in H9 hESCs treated with siControl and si*ESRG* by immunofluorescence staining. Scale bar, 20 μm. (**I** and** J**) MCM2 overexpression partially rescued morphological changes (I) and the protein levels of OCT4 and NANOG (J) reduced by *ESRG* knockdown. oe, overexpression. Scale bar, 100 μm. (**K-N**) MCM2 overexpression partially rescued the cell proliferation (K) and apoptosis (M) induced by *ESRG* knockdown. The quantified analyses of EdU and apoptosis assay are shown in (L) and (N). All representative examples of the data from at least three independent experiments are shown. Data are presented as mean ± SD. **P < 0.01 by two-tailed Student's *t* test.

We found that MCM2 protein levels were significantly reduced when *ESRG* was knocked down in both hESCs and hiPSCs (Fig. 5A). To further determine whether *ESRG* affects MCM2 stability, we treated hESCs with cycloheximide (CHX), a translation inhibitor, and found that *ESRG* knockdown markedly shortened the half-life of MCM2 protein in hESCs (Fig. 5B). Moreover, the ubiquitination of MCM2 was enhanced by *ESRG* knockdown (Fig. 5D). Then, we treated the cells with the proteasome inhibitor MG132 to prevent MCM2 degradation. As shown in Fig. 5C, the *ESRG* siRNA-induced MCM2 reduction was abolished by the proteasome inhibitor MG132. Similar results were obtained in hiPSCs after CHX and MG132 treatment (Fig. S5E-G).

Next, we investigated the mechanisms of *ESRG*-mediated inhibition of MCM2 ubiquitination and identified the factors targeting MCM2 ubiquitination. Previous studies have reported that two ubiquitin ligases, TRAIP and CRL2^LRR1^, can bind to the MCM2-7 complex to regulate DNA replication 38-40, so we speculated that they might be involved in *ESRG*-mediated MCM2 degradation. Therefore, we used siRNAs to interfere with the expression of *ESRG* and TRAIP or CRL2^LRR1^ at the same time and found that inhibition of TRAIP rather than CRL2^LRR1^ reversed MCM2 downregulation by *ESRG* siRNA (Fig. 5E and Fig. S5H). These results suggest that TRAIP can bind to MCM2 and mediate the posttranscriptional regulation of MCM2 by *ESRG*. Then, the binding of TRAIP to MCM2 was confirmed by a Co-IP assay (Fig. 5F). Importantly, *ESRG* knockdown enhanced the TRAIP/MCM2 interaction in hPSCs (Fig. 5F). Together, these data indicate that *ESRG* stabilizes MCM2 protein by interfering with the TRAIP/MCM2 interaction and inhibiting the ubiquitination of MCM2.

Based on the interaction of *ESRG* and MCM2, we examined whether *ESRG* affects the cellular localization of MCM2 as well as its expression level. Therefore, the changes in MCM2 localization were detected by assessing the nuclear and cytoplasmic fractions of hPSCs. Our results showed that knockdown of *ESRG* sequestered more MCM2 in the cytoplasm (Fig. 5G), which was also confirmed by an immunofluorescence assay (Fig. 5H and Fig. S5I). Taken together, these findings implied that *ESRG* affects the DNA replication and self-renewal of hPSCs by influencing the cellular localization of MCM2.

We next tested whether MCM2 is essential for *ESRG* function. We examined the effect of MCM2 overexpression in *ESRG* knockdown hPSCs and found that overexpression of MCM2 partially rescued the morphological changes and the reduction in clone formation ability caused by *ESRG* knockdown (Fig. 5I). Additionally, MCM2 overexpression also partially rescued *ESRG* knockdown-induced proliferation slowing and apoptosis enhancement (Fig. 5K-N). Interestingly, MCM2 not only affected the maintenance of self-renewal by *ESRG*, but also partially restored the expression of the pluripotency markers OCT4, SOX2 and NANOG (Fig. 5J). These data suggest that MCM2 is essential for *ESRG* to maintain hPSC self-renewal and pluripotency.

### *ESRG*-MCM2 maintains the self-renewal and pluripotency of hPSCs by suppressing the p53 signaling pathway

**Figure 6:**
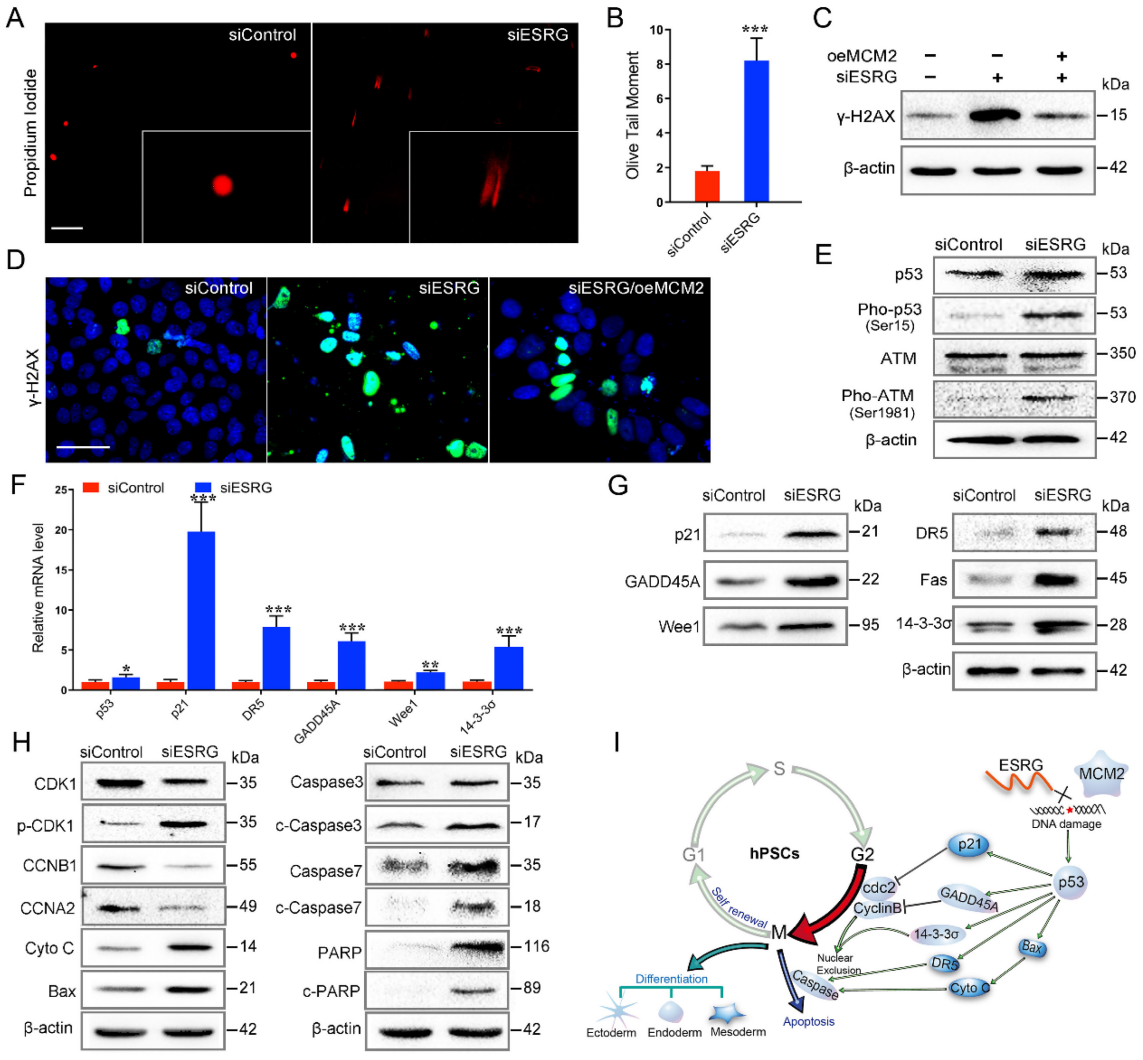
***ESRG*-MCM2 maintains the cell survival and self-renewal/pluripotency of hPSCs by suppressing the p53 signaling pathway. (A)** Representative pictures of comet assay performed 48 h in H9 hESCs after treatment with siControl and si*ESRG*. Scale bar, 200 μm.** (B)** The quantified analysis of comet assay.** (C** and** D)** H9 hESCs were transfected with MCM2 vector followed by *ESRG* knockdown. γ-H2AX protein expression was detected by Western blot (C) and immunofluorescence staining (D). Scale bar, 100 μm. **(E)** Western blot was performed to analyze the protein levels of p53, Pho-p53, ATM and Pho-ATM in H9 cells treated with siControl and si*ESRG*. **(F** and** G)** The expression of p53 signaling pathway genes was analyzed by qPCR (F) and Western blot (G) in H9 hESCs transfected with si*ESRG* or siControl. **(H)** Western blot was performed to analyze the protein levels of apoptosis-related proteins in H9 cells treated with siControl and si*ESRG*. **(I)** Schematic model of the mechanisms by which *ESRG* affects the cell survival and self-renewal/pluripotency of hPSCs. All representative examples of the data from at least three independent experiments are shown. Data are presented as mean ± SD. *P < 0.05, **P < 0.01 and ***P < 0.001 by two-tailed Student's* t* test.

MCM2 is involved in the initiation of DNA replication through the formation of the MCM2-7 complex, and the abnormalities in MCM2 and MCM2 depletion can activate a DNA damage response 38, 41, 42. To evaluate the DNA damage in hPSCs after *ESRG* knockdown, a single-cell gel electrophoresis assay (comet assay) was performed. The migration of DNA fragments released from the nucleus forms the tail, with a longer tail length and tail olive moment representing severe damage. As shown in Fig. 6A-B, the olive tail moment was significantly increased after *ESRG* knockdown compared with the control group. In addition, we found that after *ESRG* knockdown in hPSCs, γ-H2AX protein levels and foci formation increased (Fig. 6C-D and Fig. S6A-C), further confirming the induction of double strand breaks (DSBs). Importantly, this phenomenon could be partially reversed by MCM2 overexpression (Fig. 6C-D), revealing that si*ESRG* caused DNA damage through MCM2. Then, DNA damage could trigger G1/S or G2/M arrest as a protective response, giving the cells time to repair before division 43, which is consistent with our previous results (Fig. 2D-E).

It has been reported that ATM kinase is activated after DNA damage, and ATM rapidly phosphorylates p53, which then activates the p53 pathway 44, 45. Here, we detected the phosphorylation levels of ATM kinase and p53 in hPSCs after *ESRG* knockdown, and the results confirmed this hypothesis (Fig. 6E). Pathway information generated by KEGG showed that *ESRG* knockdown led to the changes in multiple signaling pathways, including the p53 pathway, DNA replication pathway, cell cycle pathway, PI3K-Akt pathway, apoptosis pathway and pathways regulating the pluripotency of stem cells (Fig. S6D and Table S2). Significant changes in the expression of many important components (*p21*,* DR5*, *14-3-3σ*, *GADD45A*, *Wee1*, etc.) of the p53 signaling pathway were observed between *ESRG* knockdown and control cells, which was also confirmed by qPCR and PCR Array of Human p53 Signaling Pathway (Fig. 6F and Fig. S6E-F). Furthermore, the protein levels of p53, p21, DR5, GADD45A and 14-3-3σ were also significantly increased in *ESRG* knockdown hPSCs (Fig. 6G).

The protein levels of the G2/M transition components of the cell cycle, including CDK1, CCNB1 and CCNA2, were significantly downregulated, and the expression levels of proapoptotic proteins, including Cyto C, BAX, Caspase3, cleaved (c)-Caspase3, Caspase7, c-Caspase7, PARP and c-PARP, were upregulated in *ESRG* knockdown hPSCs compared with the control cells (Fig. 6H and Fig. S6G). The results of *ESRG* knockdown are consistent with previously reported studies indicating that MCM2 knockdown leads to DNA damage and reduced cell proliferation 41, 42, and our further experiments showed that inhibition of p53 could partially rescue these phenomena (Fig. S6H-I). Collectively, these results suggested that *ESRG* knockdown leads to MCM2 degradation and nuclear export, resulting in DNA damage and activation of the p53 signaling pathway, thus impairing pluripotency and self-renewal of hPSCs (Fig. 6I).

**Figure 7:**
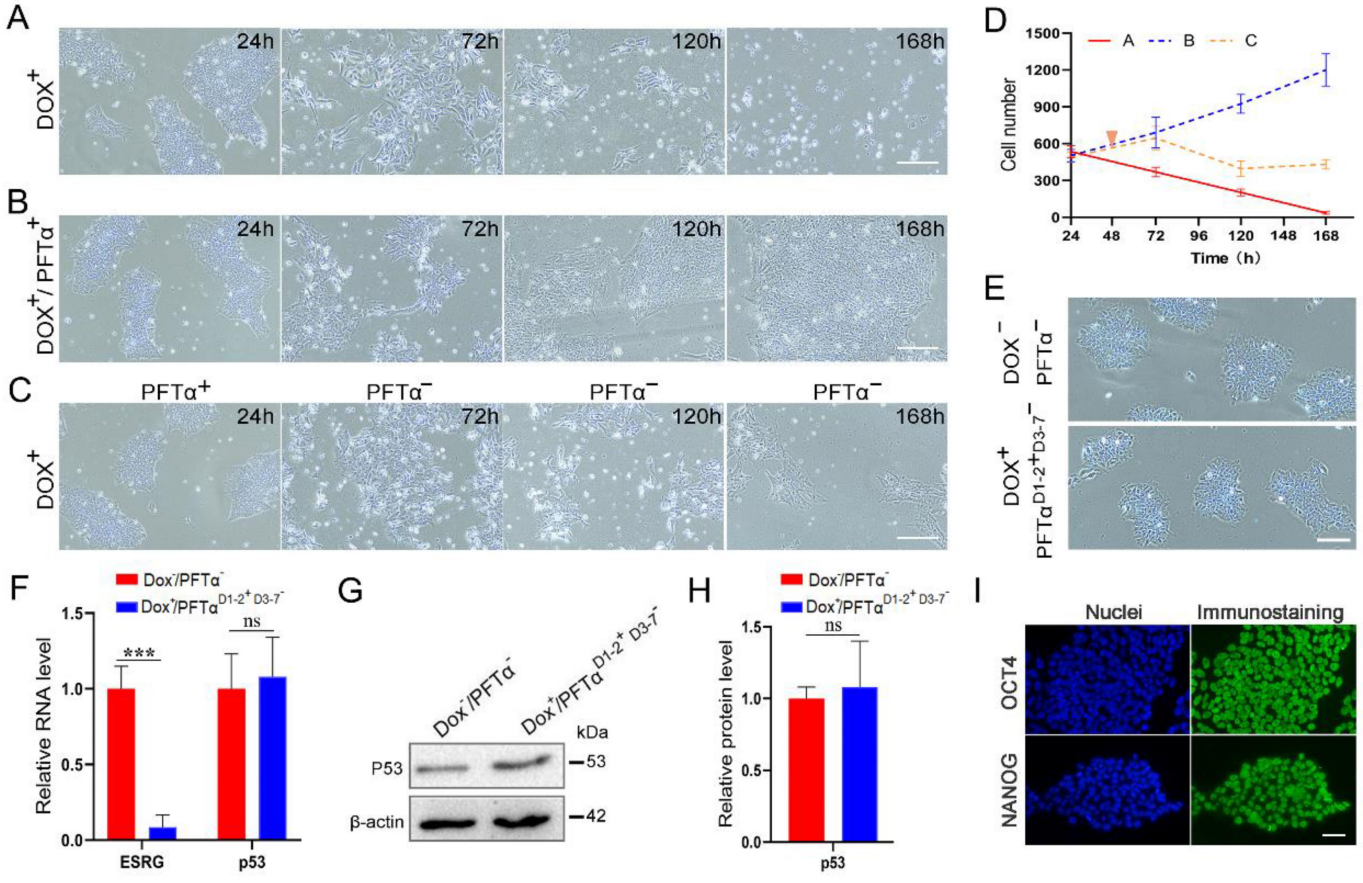
** hPSCs are sensitive to p53 in the initial period of *ESRG* knockdown. (A)** Brightfield images of H9-TetR-sh*ESRG* cells after Dox treatment for different durations. Scale bar, 100 μm. **(B)** Brightfield images of H9-TetR-sh*ESRG* cells after Dox (0.01 μg/mL) and PFTα (10 µΜ) treatment for different duration. PFTα: a p53 inhibitor. Scale bar, 100 μm. **(C)** H9-TetR-sh*ESRG* cells were treated with Dox (0.01 µg/mL) and PFTα (10 µΜ), but the addition of PFTα had stopped since day 3. Displayed are representative images of the cells captured at different durations. Scale bar, 100 μm. **(D)** The quantified analysis of cell number in (A-C). **(E)** Phase images of H9-TetR-sh*ESRG* cells under different conditions (upper panel: H9-TetR-sh*ESRG* cells under normal growth, without any treatment; lower panel: Cells in (C) continued to grow after 7 days and underwent their first passage). Scale bar, 100 μm. **(F)** qPCR detection for expression of *ESRG* and *p53* in two groups of cells in (E). **(G)** Western blotting detection for expression of p53 in two groups of cells in (E). **(H)** The quantified analysis of (G). **(I)** The expression of OCT4 and NANOG in surviving cells (C) was detected by immunofluorescence assay. Scale bar, 100 μm. Data are presented as mean ± SD. ***P < 0.001 by two-tailed Student's *t* test, ns=no significance.

However, a recent study by Takahashi, et al., demonstrated *ESRG* is dispensable when *ESRG*-knockout hPSC lines are generated under p53 siRNA treatment 20. To explain the seemingly contradictory observations, we hypothesize that temporarily knocking down p53 during the construction of *ESRG* knockout cell lines may have led to the different results. We tested the hypothesis with H9-TetR-sh*ESRG* inducible cell line (Fig. 7). When *ESRG* silencing was induced with Dox without p53 inhibition, the number of dead cells increased significantly, and almost all the cells died by day 7 (Fig. 7A and 7D). However, Dox-induced cells grew normally under continuous treatment with p53 inhibitor PFTα (Fig. 7B and 7D). After p53 inhibition was stopped on Day 3, severe cell death was observed again, however, some cells survived with normal stem cell morphology (Fig. 7C and 7D). We cultured and passaged the surviving cells and found that the cell morphology was consistent with normal hPSCs (Fig. 7E). Then we collected cells for a series of tests. qPCR and western blot results showed that compared with normal cells, the expression of *ESRG* was extremely low while p53 expression was normal (Fig. 7F-H). Immunofluorescence assay also showed the normal expression of OCT4 and NANOG (Fig. 7I). These results suggest that hPSCs can survive when *ESRG* is knocked down with p53 inhibition, which agrees with the report by Takahashi, et al. 20.

## Discussion

*ESRG,* which was identified in our previous studies, exists only in human and some primate cells and is highly expressed only in hPSCs, suggesting that *ESRG* has unique significance in hPSCs*.* Wang, et al., found that *ESRG* is required for the maintenance of the self-renewal and pluripotency of hESCs, as knockdown of *ESRG* leads to the differentiation of H9 hESCs 19. Furthermore, the studies of Rand et al. and Sekine et al. also support our results 17, 18. However, Takahashi, et al., showed that *ESRG* is dispensable for human pluripotency, which causes a great discrepancy 20. This paper has addressed the controversy from a variety of aspects.

We demonstrated that a decrease in *ESRG* expression caused a drastic change in cell and colony morphology, with the loss of several stem cell markers, including OCT4, SOX2 and NANOG. hESCs and hiPSCs changed into scattered, long and thin differentiated cells with a slow proliferation rate when *ESRG* expression was downregulated. Recent studies have clearly shown that the self-renewal and differentiation of stem cells are closely connected to cell cycle progression 46, 47. OCT4, SOX2, NANOG, JMJD5, p53 and the RB family 48-50 control the self-renewal and/or pluripotent status of hPSCs by cell cycle regulation. Our data showed that *ESRG* also plays an important role in regulating the cell cycle progression of hPSCs. Specific repression of *ESRG* in hPSCs caused the accumulation of cells in the G2/M phase and resulted in increased apoptosis and decreased cell proliferation. We used different siRNA sequences and obtained consistent results. Rescue experiments also showed that there were no off-target effects. Then, we used BLAST and other tools to compare siRNA sequences, and all genes that might be affected were detected by qPCR to exclude possible off-target effects. In addition, several attempts were made to construct *ESRG* knockout cell line by using CRISPR technology without p53 knockdown, but none of them were successful, which further demonstrates the importance of *ESRG* for hPSCs. In fact, studies have shown that *ESRG* can serve as a pluripotency marker, is turned on as early as day 3 after reprogramming induced by OCT3/4, SOX2 and KLF4 (OSK), and can be used as a marker for early reprogramming of cells and undifferentiated hPSCs 17, 18. These studies also strongly support our results regarding the essential role of *ESRG* in hPSCs.

OCT4, SOX2 and NANOG form a regulatory feedback circuit to maintain pluripotency in PSCs 3, 51. In this circuit, all three transcription factors regulate themselves and each other. This regulatory circuit is essential to PSC identity 11, 52. Previous bioinformatics analysis predicted an OCT4 binding site upstream of the *ESRG* gene, suggesting that *ESRG* may be regulated by OCT4 13. Our results showed that *ESRG* is a downstream target of OCT4 in hPSCs. We thus concluded that the interaction of OCT4 with the regulatory region of *ESRG* serves to enhance the transcription of *ESRG*. *ESRG* is involved in the OCT4, SOX2 and NANOG transcriptional regulation network.

LncRNAs usually function in stem cells by binding to proteins 53-56. Here, seventy-two proteins specifically bound to *ESRG* were screened out by RNA pull-down and mass spectrometry. After further experimental verification, we identified that *ESRG* bound to MCM2, a replication-licensing factor, to sustain its steady-state level and nuclear location. MCM2, as a member of the MCM family of proteins, encodes a protein of 904 amino acids containing a presumptive zinc-finger domain 57. It forms a double hexamer with MCM3-7 and loads onto chromatin with the origin recognition complex, Cdc6 and Cdt1, to form replication complexes and initiate DNA replication 58. The importance of MCM2 in DNA replication and cell proliferation makes it a highly specific and sensitive marker, which has diagnostic and prognostic significance in various human malignant tumors 59-63. As an indispensable member of MCM2-7, MCM2 plays a significant role in DNA replication, and the loss of MCM2 can inhibit cell proliferation 64-66 and lead to cell death due to DNA damage 41, 67, 68.

In response to DNA damage, p53 maintains genomic stability by regulating the expression of its downstream target genes (*p21*, *GADD45A*, *14-3-3σ* and *DR5*), which are involved in apoptosis, the cell cycle, cell proliferation, cell differentiation, and DNA repair 69-70. p53 expression is induced during hPSC differentiation, and a highly undifferentiated state and regenerative potential of hPSCs can be obtained by suppressing p53 8. In this study, we showed that *ESRG* knockdown activated the p53 signaling pathway, leading to cell cycle arrest, reduced proliferation, and increased apoptosis and differentiation of hPSCs, and these results are similar to the effects of knockdown of OCT4, Rem2 and Aurka in hPSCs 71, 72. Thus, our results suggested that *ESRG* knockdown leads to MCM2 degradation and nuclear export, resulting in DNA damage and the activation of the p53 signaling pathway, leading to cell proliferation slowing, cell cycle arrest and apoptosis, thus impairing the cell survival and self-renewal/pluripotency of hPSCs (Fig. 6I).

Our findings of *ESRG*'s essential roles are consistent with a few previous reports. However, a recent study by Takahashi, et al., demonstrated *ESRG* is dispensable when *ESRG*-knockout hPSC lines are generated under p53 siRNA treatment 20. Through our experiments, we found that hPSCs can survive when *ESRG* is knocked down with p53 inhibition, which agrees with the report by Takahashi, et al. Based on these results, we concluded that the hPSCs are extremely sensitive to p53 when *ESRG* is knocked down, and once the cells tolerate it, the cells will no longer be affected by p53. Although p53 inhibition is temporary, the effect is critical to prevent acute cell death caused by the loss of *ESRG*. As for why p53 is so important for *ESRG* knockdown effects and how the cells adapt after a few days, it awaits further investigation.

In addition to MCM2, there are other proteins that bind to *ESRG* with high affinity, such as PSMD11, PODXL and NPM1. Although these proteins have not been shown to play a role in hPSCs by interacting with *ESRG*, several studies have shown that they are important for self-renewal and maintenance of pluripotency in stem cells. For example, hPSCs have higher proteasome activity than differentiated cells, which is induced by PSMD11/RPN6, a scaffold subunit that promotes proteasome assembly 73, 74. Undifferentiated hPSCs can be isolated using a specific recognition antibody against PODXL, which can also be used in stem cell therapy and has been patented. NPM1 is an essential gene for embryonic development and the proliferation of embryonic stem cells 75, and is closely related to the proliferation and apoptosis of neural stem cells 76. These studies suggest that in addition to maintaining the proliferation and self-renewal of hPSCs by binding to MCM2, *ESRG* combines with other proteins to participate in the vital activities of hPSCs.

Overall, this study identified a clear role of *ESRG* in maintaining cell survival of hPSCs. We expanded on the significance of *ESRG* as a target of the stem cell regulatory factor OCT4 in hPSCs and demonstrated that in hPSCs, *ESRG* binds with MCM2 to sustain the steady-state level and nuclear location of MCM2, inhibit cell differentiation, promote cell proliferation and inhibit cell apoptosis by suppressing p53 signaling. These findings contribute to our understanding of the molecular mechanisms of self-renewal and pluripotency maintenance of hPSCs and provide insights for further biological study and the development of promising clinical applications of *ESRG* in stem cell therapy.

## Supplementary Material

Supplementary figures and tables.

## Abbreviations

AP: alkaline phosphatase
bFGF: basic fibroblast growth factor
ChIP: chromatin immunoprecipitation
CHX: cyclohexamide
CM: conditioned medium
Co-IP: co-immunoprecipitation
Dox: doxycycline
DSBs: double strand breaks
E8 medium: essential 8 medium
hESCs: human embryonic stem cells
hiPSCs: induced pluripotent stem cells
hPSCs: human pluripotent stem cells
IF-FISH: immunofluorescence-FISH
ISH: *in situ* hybridization
lncRNAs: long non-coding RNAs
MCM2: minichromosome maintenance 2
NSG: NOD-PrkdcscidIL2rgem2/SMOC
OSK: OCT3/4, SOX2 and KLF4
RIP: RNA immunoprecipitation
RT: room temperature
SD: standard deviation
SPF: specific pathogen free

## References

[1] Thomson JA, Itskovitz-Eldor J, Shapiro SS (1998). Embryonic stem cell lines derived from human blastocysts. Science.

[2] Zakrzewski W, Dobrzynski M, Szymonowicz M (2019). Stem cells: past, present, and future. Stem Cell Res Ther.

[3] Wang Z, Oron E, Nelson B (2012). Distinct lineage specification roles for NANOG, OCT4, and SOX2 in human embryonic stem cells. Cell Stem Cell.

[4] Gifford CA, Ziller MJ, Gu H (2013). Transcriptional and epigenetic dynamics during specification of human embryonic stem cells. Cell.

[5] Warmflash A, Arduini BL, Brivanlou AH (2012). The molecular circuitry underlying pluripotency in embryonic stem cells. Wiley Interdiscip Rev Syst Biol Med.

[6] Tsankov AM, Gu H, Akopian V (2015). Transcription factor binding dynamics during human ES cell differentiation. Nature.

[7] Xu N, Papagiannakopoulos T, Pan G (2009). MicroRNA-145 regulates OCT4, SOX2, and KLF4 and represses pluripotency in human embryonic stem cells. Cell.

[8] Zhang ZN, Chung SK, Xu Z (2014). Oct4 maintains the pluripotency of human embryonic stem cells by inactivating p53 through Sirt1-mediated deacetylation. Stem Cells.

[9] Simandi Z, Horvath A, Wright LC (2016). OCT4 acts as an integrator of pluripotency and signal-induced differentiation. Mol Cell.

[10] Orkin SH (2005). Chipping away at the embryonic stem cell network. Cell.

[11] Boyer LA, Lee TI, Cole MF (2005). Core transcriptional regulatory circuitry in human embryonic stem cells. Cell.

[12] Hosseinpour B, Bakhtiarizadeh MR, Khosravi P (2013). Predicting distinct organization of transcription factor binding sites on the promoter regions: a new genome-based approach to expand human embryonic stem cell regulatory network. Gene.

[13] Zhao M, Ren C, Yang H (2007). Transcriptional profiling of human embryonic stem cells and embryoid bodies identifies H*ESRG*, a novel stem cell gene. Biochem Biophys Res Commun.

[14] Wanggou S, Jiang X, Li Q (2012). H*ESRG*: a novel biomarker for intracranial germinoma and embryonal carcinoma. J Neurooncol.

[15] Jafari N, Najafabadi AN, Hamzei B (2021). *ESRG*, LINC00518 and PWRN1 are newly-identified deregulated lncRNAs in colorectal cancer. Exp Mol Pathol.

[16] Filippov-Levy N, Cohen-Schussheim H, Trope CG (2018). Expression and clinical role of long non-coding RNA in high-grade serous carcinoma. Gynecol Oncol.

[17] Rand TA, Sutou K, Tanabe K (2018). MYC releases early reprogrammed human cells from proliferation pause via retinoblastoma protein inhibition. Cell Rep.

[18] Sekine K, Tsuzuki S, Yasui R (2020). Robust detection of undifferentiated iPSC among differentiated cells. Sci Rep.

[19] Wang J, Xie G, Singh M (2014). Primate-specific endogenous retrovirus-driven transcription defines naive-like stem cells. Nature.

[20] Takahashi K, Nakamura M, Okubo C (2021). The pluripotent stem cell-specific transcript *ESRG* is dispensable for human pluripotency. PLoS Genet.

[21] Ren C, Zhao M, Yang X (2006). Establishment and applications of epstein-barr virus-based episomal vectors in human embryonic stem cells. Stem Cells.

[22] Liu H, Ren C, Zhu B (2016). High-efficient transfection of human embryonic stem cells by single-cell plating and starvation. Stem Cells Dev.

[23] Liu H, Zhang Y, Zhang YY (2020). Human embryonic stem cell-derived organoid retinoblastoma reveals a cancerous origin. Proc Natl Acad Sci U S A.

[24] Zhao M, Yang H, Jiang X Lipofectamine RNAiMAX: an efficient siRNA transfection reagent in human embryonic stem cells, Mol Biotechnol. 2008; 40: 19-26.

[25] Tulpule A, Lensch MW, Miller JD (2010). Knockdown of Fanconi anemia genes in human embryonic stem cells reveals early developmental defects in the hematopoietic lineage. Blood.

[26] Zaehres H, Lensch MW, Daheron L (2005). High-efficiency RNA interference in human embryonic stem cells. Stem Cells.

[27] Zafarana G, Avery SR, Avery K (2009). Specific knockdown of OCT4 in human embryonic stem cells by inducible short hairpin RNA interference. Stem Cells.

[28] Liu H, Li S, Ren C (2020). Generation of an *ESRG* Pr-tdTomato reporter human embryonic stem cell line, CSUe011-A, using CRISPR/Cas9 editing. Stem Cell Res.

[29] Feng X, Ren C, Zhou W (2014). Promoter hypermethylation along with LOH, but not mutation, contributes to inactivation of DLC-1 in nasopharyngeal carcinoma. Mol Carcinog.

[30] Liu J, Huang W, Ren C (2015). Flotillin-2 promotes metastasis of nasopharyngeal carcinoma by activating NF-kappaB and PI3K/Akt3 signaling pathways. Sci Rep.

[31] Jia WT, Ren CP, Wang L (2016). CD109 is identified as a potential nasopharyngeal carcinoma biomarker using aptamer selected by cell-SELEX. Oncotarget.

[32] Wang P, Xue Y, Han Y (2014). The STAT3-binding long noncoding RNA lnc-DC controls human dendritic cell differentiation. Science.

[33] You X, Vlatkovic I, Babic A (2015). Neural circular RNAs are derived from synaptic genes and regulated by development and plasticity. Nat Neurosci.

[34] Bouma MJ, van Iterson M, Janssen B (2017). Differentiation-defective human induced pluripotent stem cells reveal strengths and limitations of the teratoma assay and *in vitro* pluripotency assays. Stem Cell Reports.

[35] Ryu S, Driever W (2006). Minichromosome maintenance proteins as markers for proliferation zones during embryogenesis. Cell Cycle.

[36] Wang Y, Chen H, Zhang J (2020). MCM family in gastrointestinal cancer and other malignancies: From functional characterization to clinical implication. Biochim Biophys Acta Rev Cancer.

[37] Remus D, Beuron F, Tolun G (2009). Concerted loading of Mcm2-7 double hexamers around DNA during DNA replication origin licensing. Cell.

[38] Sedlackova H, Rask MB, Gupta R (2020). Equilibrium between nascent and parental MCM proteins protects replicating genomes. Nature.

[39] Wu RA, Semlow DR, Kamimae-Lanning AN (2019). TRAIP is a master regulator of DNA interstrand crosslink repair. Nature.

[40] Akopian D, Rape M (2017). Conducting the finale of DNA replication. Genes Dev.

[41] Bailis JM, Forsburg SL (2004). MCM proteins: DNA damage, mutagenesis and repair. Curr Opin Genet Dev.

[42] Natsume T, Nishimura K, Minocherhomji S (2017). Acute inactivation of the replicative helicase in human cells triggers MCM8-9-dependent DNA synthesis. Genes Dev.

[43] Barshishat S, Elgrably-Weiss M, Edelstein J (2018). OxyS small RNA induces cell cycle arrest to allow DNA damage repair. EMBO J.

[44] Bakkenist CJ, Kastan MB (2003). DNA damage activates ATM through intermolecular autophosphorylation and dimer dissociation. Nature.

[45] Bhatti S, Kozlov S, Farooqi AA (2011). ATM protein kinase: the linchpin of cellular defenses to stress. Cell Mol Life Sci.

[46] Gonzales KA, Liang H, Lim YS (2015). Deterministic restriction on pluripotent state dissolution by cell-cycle pathways. Cell.

[47] Pauklin S, Vallier L (2013). The cell-cycle state of stem cells determines cell fate propensity. Cell.

[48] Conklin JF, Baker J, Sage J (2012). The RB family is required for the self-renewal and survival of human embryonic stem cells. Nat Commun.

[49] Lee J, Go Y, Kang I (2010). Oct-4 controls cell-cycle progression of embryonic stem cells. Biochem J.

[50] Jain AK, Allton K, Iacovino M (2012). p53 regulates cell cycle and microRNAs to promote differentiation of human embryonic stem cells. PLoS Biol.

[51] Adachi K, Suemori H, Yasuda SY (2010). Role of SOX2 in maintaining pluripotency of human embryonic stem cells. Genes Cells.

[52] Tay Y, Zhang J, Thomson AM (2008). MicroRNAs to Nanog, Oct4 and Sox2 coding regions modulate embryonic stem cell differentiation. Nature.

[53] Zhao C, Xie W, Zhu H (2022). LncRNAs and their RBPs: How to influence the fate of stem cells?. Stem Cell Res Ther.

[54] Xie W, Zhu H Zhao M (2021). Crucial roles of different RNA-binding hnRNP proteins in stem cells. Int J Biol Sci.

[55] Liu W, Wang K, Lv X (2020). Up-regulation of RNA binding proteins contributes to folate deficiency-induced neural crest cells dysfunction. Int J Biol Sci.

[56] Zhu Y, Yan Z, Du Z (2020). Osblr8 orchestrates intrachromosomal loop structure required for maintaining stem cell pluripotency. Int J Biol Sci.

[57] Yan H, Gibson S, Tye BK (1991). Mcm2 and Mcm3, two proteins important for ARS activity, are related in structure and function. Genes Dev.

[58] Shechter D, Ying CY, Gautier J (2004). DNA unwinding is an Mcm complex-dependent and ATP hydrolysis-dependent process. J Biol Chem.

[59] Reena RM, Mastura M, Siti-Aishah MA (2008). Minichromosome maintenance protein 2 is a reliable proliferative marker in breast carcinoma. Ann Diagn Pathol.

[60] Giaginis C, Vgenopoulou S, Vielh P (2010). MCM proteins as diagnostic and prognostic tumor markers in the clinical setting. Histol Histopathol.

[61] Giaginis C, Georgiadou M, Dimakopoulou K (2009). Clinical significance of MCM-2 and MCM-5 expression in colon cancer: association with clinicopathological parameters and tumor proliferative capacity. Dig Dis Sci.

[62] Sun M, Wu G, Li Y (2010). Expression profile reveals novel prognostic biomarkers in hepatocellular carcinoma. Front Biosci (Elite Ed).

[63] Quaglia A, McStay M, Stoeber K (2006). Novel markers of cell kinetics to evaluate progression from cirrhosis to hepatocellular carcinoma. Liver Int.

[64] Maslov AY, Bailey KJ, Mielnicki LM (2007). Stem/progenitor cell-specific enhanced green fluorescent protein expression driven by the endogenous Mcm2 promoter. Stem Cells.

[65] Kong S, Han X, Cui T (2016). MCM2 mediates progesterone-induced endometrial stromal cell proliferation and differentiation in mice. Endocrine.

[66] Liu F, Yuan JH, Huang JF (2016). Long noncoding RNA FTX inhibits hepatocellular carcinoma proliferation and metastasis by binding MCM2 and miR-374a. Oncogene.

[67] Stead BE, Brandl CJ, Davey MJ (2011). Phosphorylation of Mcm2 modulates Mcm2-7 activity and affects the cell's response to DNA damage. Nucleic Acids Res.

[68] Kunnev D, Rusiniak ME, Kudla A (2010). DNA damage response and tumorigenesis in Mcm2-deficient mice. Oncogene.

[69] Vousden KH, Prives C (2009). Blinded by the light: the growing complexity of p53. Cell.

[70] Lin T, Hou PF, Meng S (2019). Emerging roles of p53 related lncRNAs in cancer progression: a systematic review. Int J Biol Sci.

[71] Edel MJ, Menchon C, Menendez S (2010). Rem2 GTPase maintains survival of human embryonic stem cells as well as enhancing reprogramming by regulating p53 and cyclin D1. Genes Dev.

[72] Lee DF, Su J, Ang YS (2012). Regulation of embryonic and induced pluripotency by aurora kinase-p53 signaling. Cell Stem Cell.

[73] Vilchez D, Boyer L, Morantte I (2012). Increased proteasome activity in human embryonic stem cells is regulated by PSMD11. Nature.

[74] Pathare GR, Nagy I, Bohn S (2012). The proteasomal subunit Rpn6 is a molecular clamp holding the core and regulatory subcomplexes together. Proc Natl Acad Sci U S A.

[75] Wang BB, Lu R, Wang WC (2006). Inducible and reversible suppression of Npm1 gene expression using stably integrated small interfering RNA vector in mouse embryonic stem cells. Biochem Biophys Res Commun.

[76] Qing Y, Yingmao G, Lujun B (2008). Role of Npm1 in proliferation, apoptosis and differentiation of neural stem cells. J Neurol Sci.

