# Supplementary material for: ESRG is critical to maintain the cell survival and self-renewal/pluripotency of hPSCs by collaborating with MCM2 to suppress p53 pathway
Source: Int J Biol Sci. 2023 Jan 16;19(3):916–35. doi: 10.7150/ijbs.79095 (PMC9909993; doi:10.7150/ijbs.79095)
Supplement: Supplementary file 1 — Supplementary figures and tables. [file ijbsv19p0916s1.pdf]

Supplementary materials

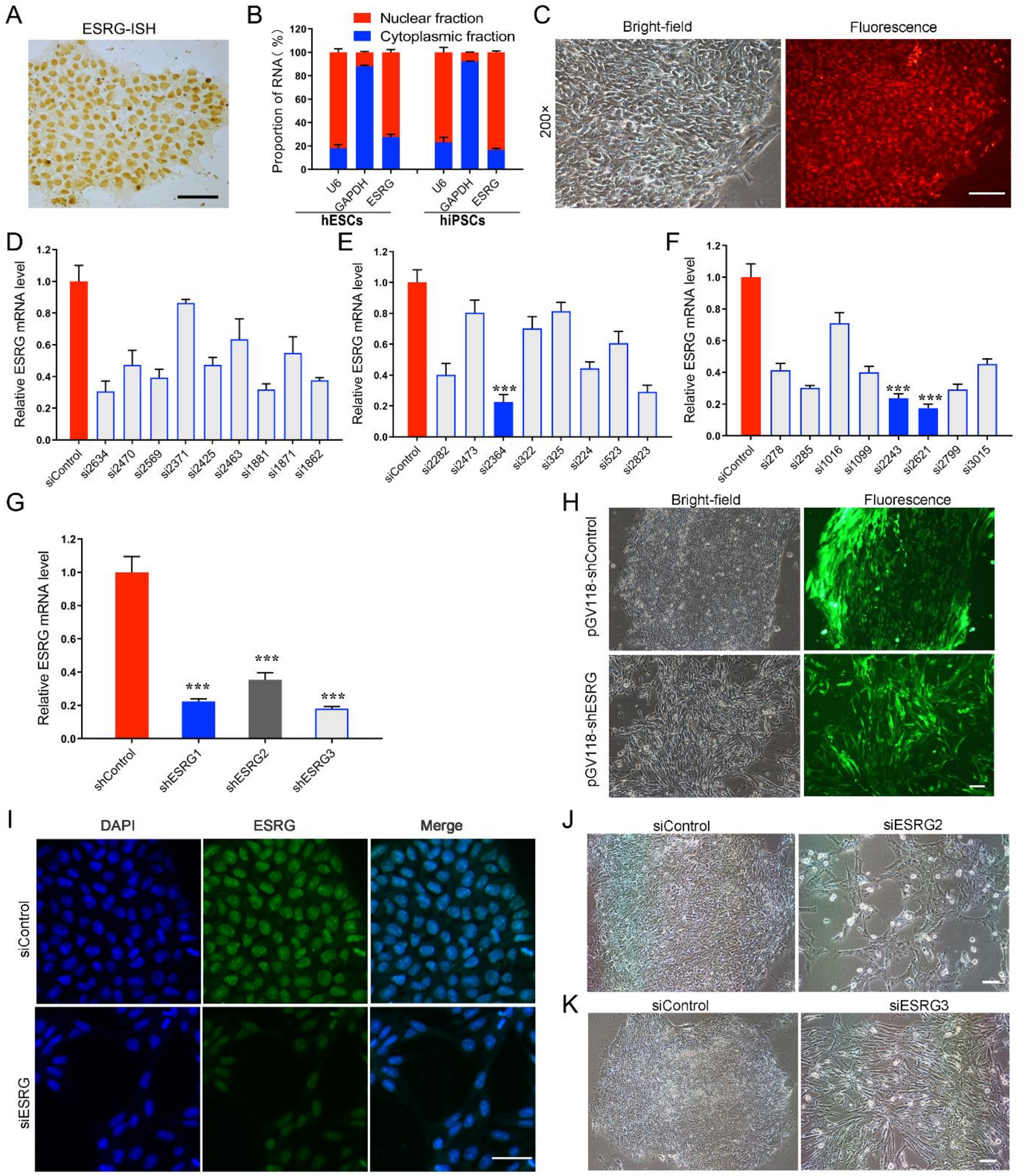

Fig. S1. The characterization of *ESRG*.

(A) *ESRG* intracellular localization was visualized in hPSCs by ISH assays. Representative image of *ESRG* (DIG) in RC1 hiPSCs is shown. Scale bar, 100 μm. **(B)** The *ESRG* expression levels in the nuclear and cytoplasmic fractions derived from H9 hESCs and RC1-iPSCs. **(C)**

Fluorescent microscope images of H9 hESCs transfected with Cy3-labeled negative siRNA. Scale bar, 100 μm. **(D-F)** QPCR analysis of the *ESRG* mRNA level in H9 hESCs transfected with twenty-five different sets of siRNAs against *ESRG*. **(G)** QPCR analysis of *ESRG* mRNA levels in H9 hESCs infected with lentivirus carrying the control vector, sh*ESRG*1, sh*ESRG*2 and sh*ESRG*3 at day 5. (**H)** Bright-field (left) and fluorescence (right) microscopy of H9 hESCs infected with the pGV118-sh*ESRG*1 (lower panel) or pGV118-shControl (upper panel) lentivirus at day 5. Green fluorescence represents cells successfully infected with lentivirus. Scale bar, 100 μm. **(I)** *ESRG* intracellular localization was visualized in H9 hESCs transfected with siControl or si*ESRG* by RNA-FISH assays. Representative images of *ESRG* (green) in H9 cells are shown. Nuclei were stained with DAPI. Scale bar, 100 μm. **(J** and **K)** Phase images of cells transfected with si*ESRG*2/si*ESRG*3 or siControl were observed by inverted phase microscopy. Scale bar, 100 μm. Data are presented as mean ± SD. ***$P < 0.001$ (two-tailed Student's *t* test).

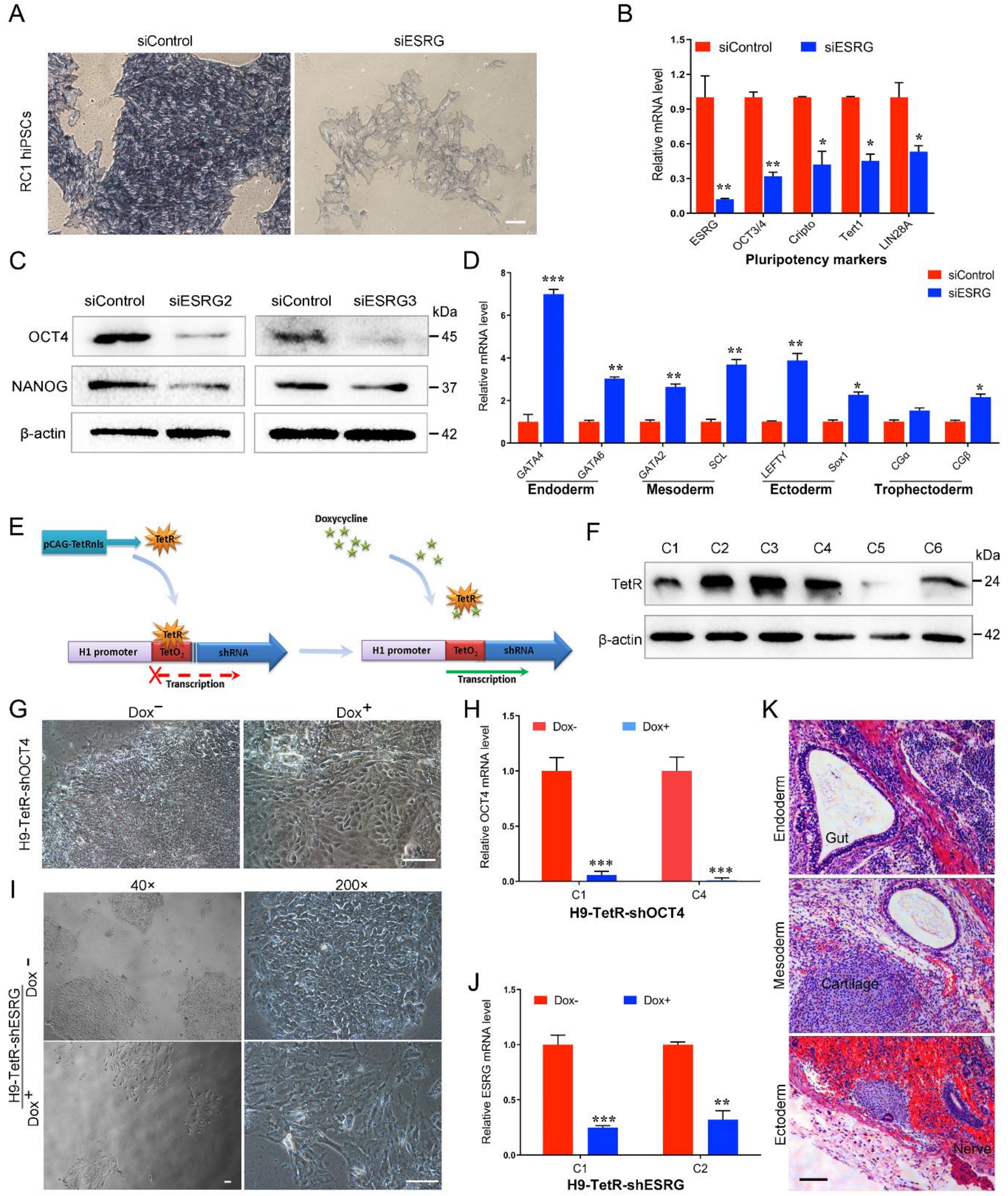

Fig. S2. *ESRG* maintains pluripotency of hPSCs.

(A) AP staining was performed in RC1 hiPSCs transfected with si*ESRG* or siControl, and the dark blue color indicated undifferentiated, AP-positive cells. Scale bar, 100 μm. **(B)** The expression of pluripotency marker genes was performed by qPCR in RC1 hiPSCs transfected with si*ESRG* or siControl. **(C)** Protein levels of OCT4 and NANOG were detected by

Western blot analysis after transfected with si*ESRG*2/si*ESRG*3 and siControl in H9 hESCs. **(D)** The expression of endoderm, mesoderm, ectoderm and trophectoderm marker genes was analyzed by qPCR in RC1 hiPSCs transfected with si*ESRG*. **(E)** Schematic diagram of the Tet-inducible shRNA interference system. Cells co-transfected with Tet repressor (TetR protein) expression vector pCAG-TetRnls and tetracycline-regulated pSUPERIOR vector can constitutively express TetR protein, which binds to the $TetO_2$ sequence in the H1 promoter of the pSUPERIOR and represses the transcription of shRNA. Upon addition of Dox, Dox binds to TetR and causes a conformational change that renders TetR unable to bind to the $TetO_2$ sequence, which allows transcription of the shRNA. **(F)** Western blot analysis of the expression of TetR protein in different H9-TetR subclones. **(G)** Phase images of H9-TetR-shOCT4 cells treated with or without Dox for 5 days. A morphological differentiation in Dox-treated H9-TetR-shOCT4 cells was observed. Scale bar, 100 μm. **(H)** QPCR analysis of OCT4 gene expression in two H9-TetR-shOCT4 subclones treated with or without Dox for 5 days. **(I)** Phase images of H9-TetR-sh*ESRG* cells treated with or without Dox for 5 days. A morphological differentiation in Dox-treated H9-TetR-sh*ESRG* cells was observed. Images were taken at 200× and 40× magnifications. Scale bar, 100 μm. **(J)** QPCR analysis of *ESRG* expression in four H9-TetR-sh*ESRG* subclones treated with or without Dox for 5 days. **(K)** HE staining of teratoma from H9-TetR-sh*ESRG* cells transplanted into NSG mice without Dox treatment. Data are presented as mean ± SD. $^{**}P < 0.01$, $^{***}P < 0.001$ (two-tailed Student's *t* test).

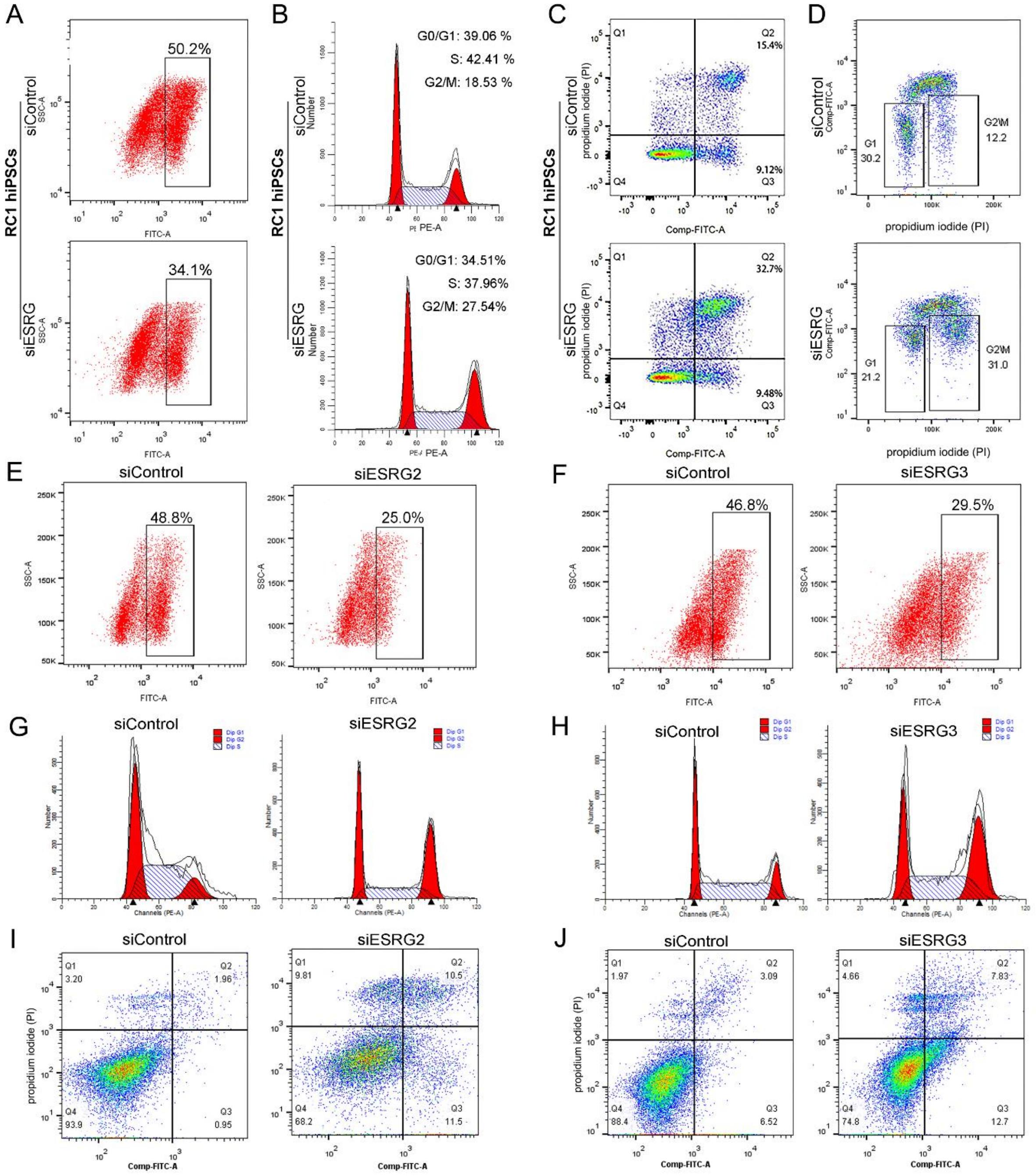

Fig. S3. ***ESRG*** **is essential for hPSC self-renewal.**

(A-C) EdU assay (A), cell cycle (B) and apoptosis assay (C) were analyzed by flow cytometry in RC1-hiPSCs transfected with si*ESRG* or siControl at 48 h after transfection**. (D)** The cell cycle distribution of H9 hESCs transfected with si*ESRG* or siControl was analyzed via flow cytometry with EdU/PI staining. **(E-J)** EdU assay (E and F), cell cycle (G and H) and apoptosis assay (I and J) were performed by flow cytometry in H9 hESCs transfected

with si*ESRG*2/si*ESRG*3 or siControl at 48 h after transfection.

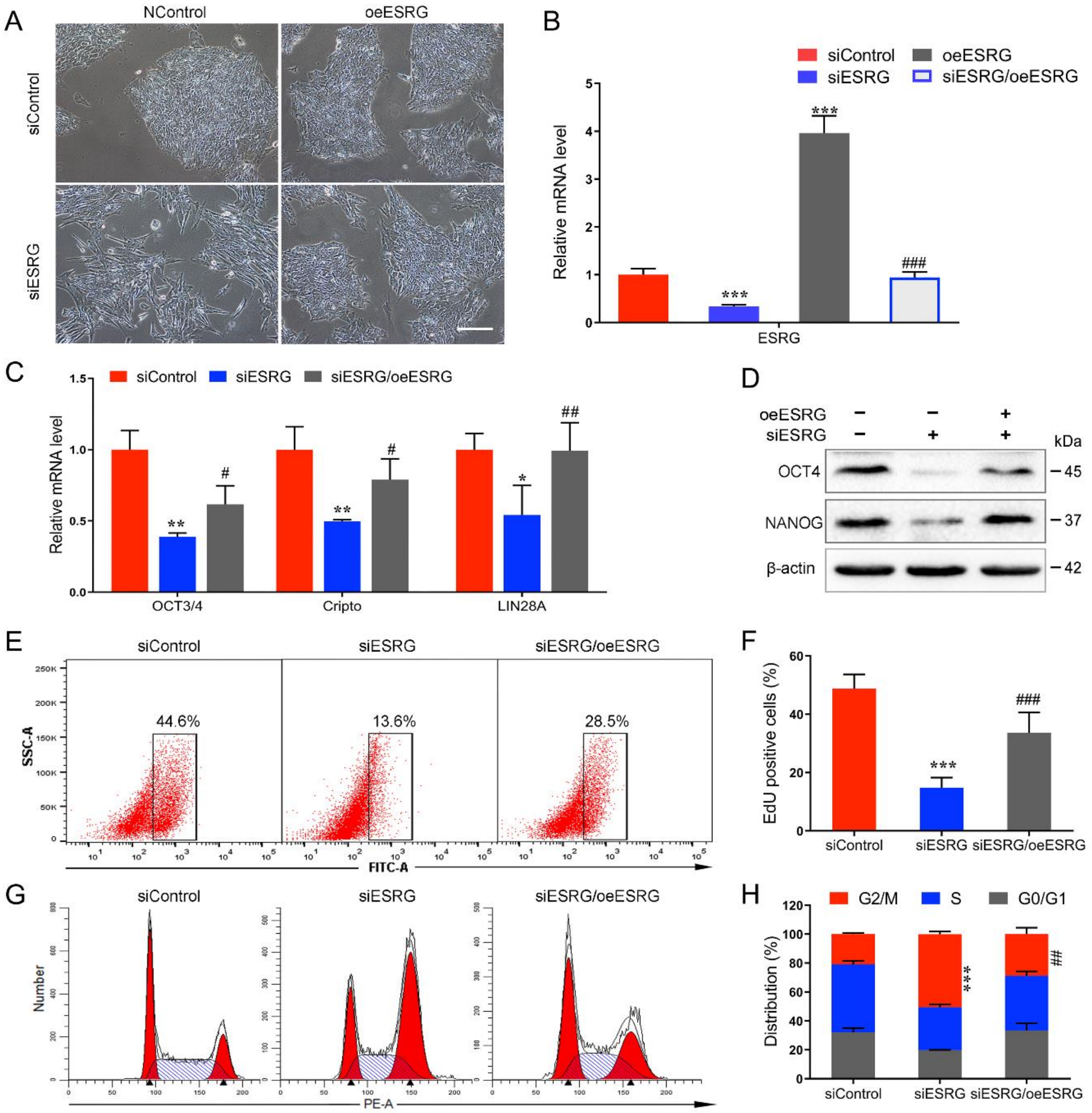

Fig. S4. Overexpression of *ESRG* rescued the morphological changes caused by *ESRG* knockdown and reversed the loss of pluripotency and the inhibition of cell proliferation.

(A) *ESRG* overexpression rescued the morphological changes induced by *ESRG* knockdown. oe, overexpression. Scale bar, 100 μm. **(B)** H9 hESCs were treated with *ESRG* adenovirus after *ESRG* knockdown, and the mRNA expression level of *ESRG* was detected by qPCR. (**C** and **D)** *ESRG* overexpression rescued the mRNA (C) and protein levels (D) of pluripotency marker genes reduced by *ESRG* knockdown. Scale bar, 100 μm. **(E-H)** *ESRG* overexpression rescued the altered cell proliferation (E) and cell cycle distribution (G) induced by *ESRG*

knockdown. The quantified analysis of EdU incorporation and cell cycle distribution are shown in (F) and (H). Data are presented as mean ± SD. *$P < 0.05$, **$P < 0.01$, ***$P < 0.001$ (si*ESRG* group versus siControl group) and #$P < 0.05$, ##$P < 0.01$, ###$P < 0.001$ (si*ESRG*/oeMCM2 group versus si*ESRG* group) (two-tailed Student's *t* test).

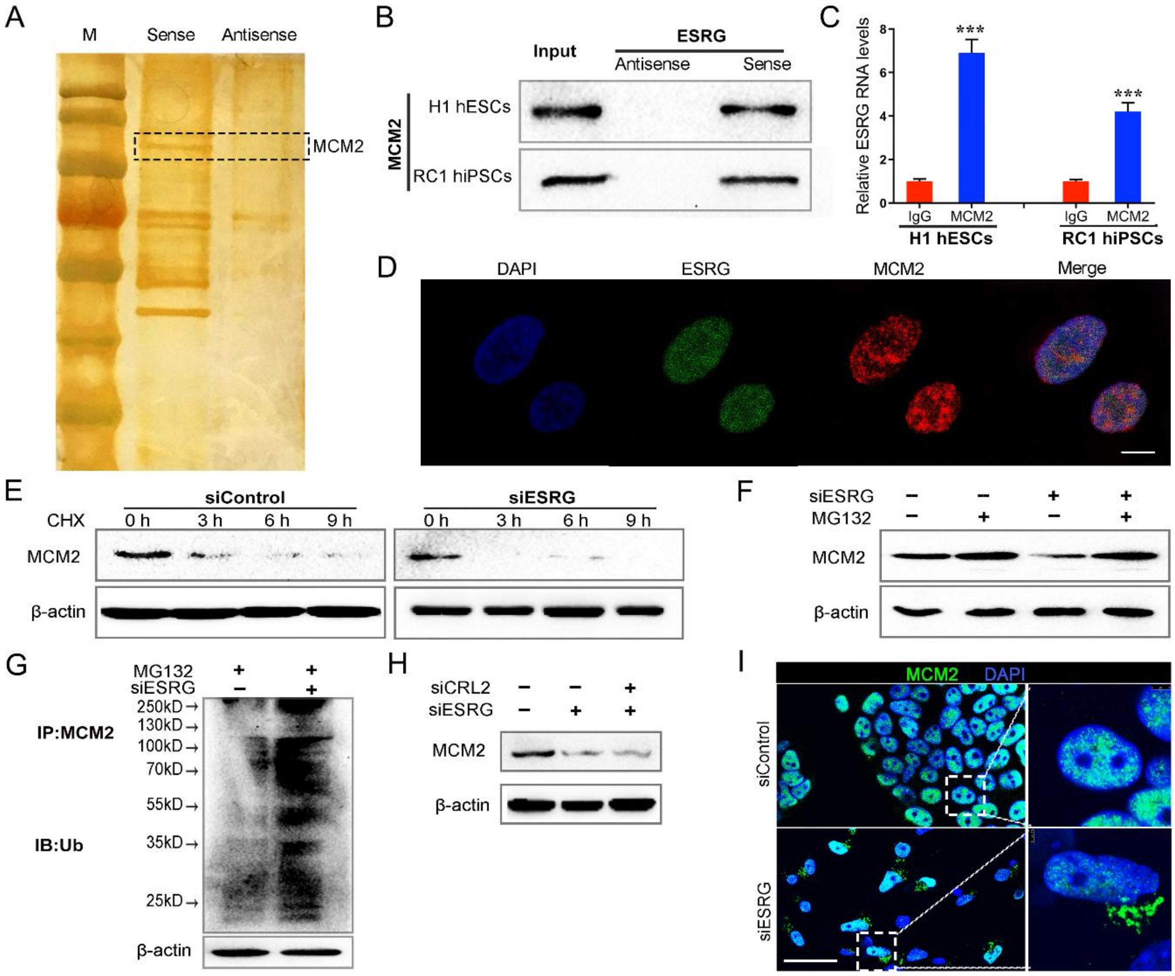

Fig. S5. *ESRG* directly binds with MCM2 in hPSCs.

(A) Biotin-RNA pulldown was performed with protein extracts of hESCs using full-length *ESRG* transcript (sense) and antisense RNA, followed by mass spectrometry. **(B and C)** The interaction of *ESRG* and MCM2 was detected through RNA pulldown (B) and RIP assays (C) in H1 hESCs and RC1-hiPSCs. **(D)** *ESRG* was visualized by RNA-FISH, and MCM2 by immunofluorescence staining in H9 hESCs was performed. Scale bar, 100 µm. **(E)** The expression of MCM2 in RC1-hiPSCs (siControl vs si*ESRG*) was detected by Western blot after treatment with CHX (20 µg/mL) for various time periods respectively. **(F)** RC1-hiPSCs were transfected with *ESRG* siRNA and pre-incubated with MG-132 (20 µM) for 4 h. Cell lysate was immunoblotted by anti-MCM2. **(G)** RC1-hiPSCs were pre-incubated with MG-132 (20 µM) for 4h. MCM2 was immunoprecipitated (IP) and immunoblotted (IB) by anti-Ub. The ubiquitination of MCM2 protein was detected after *ESRG* knockdown. **(H)** H9

cells were treated with CRL2 siRNA followed by *ESRG* knockdown. MCM2 protein expression was detected by Western blot. **(I)** MCM2 was visualized in RC1-hiPSCs treated with siControl and si*ESRG* by immunofluorescence staining. Scale bar, 20 μm. Data are presented as mean ± SD. **P* < 0.05, ***P* < 0.01, ****P* < 0.001 (two-tailed Student's *t* test).

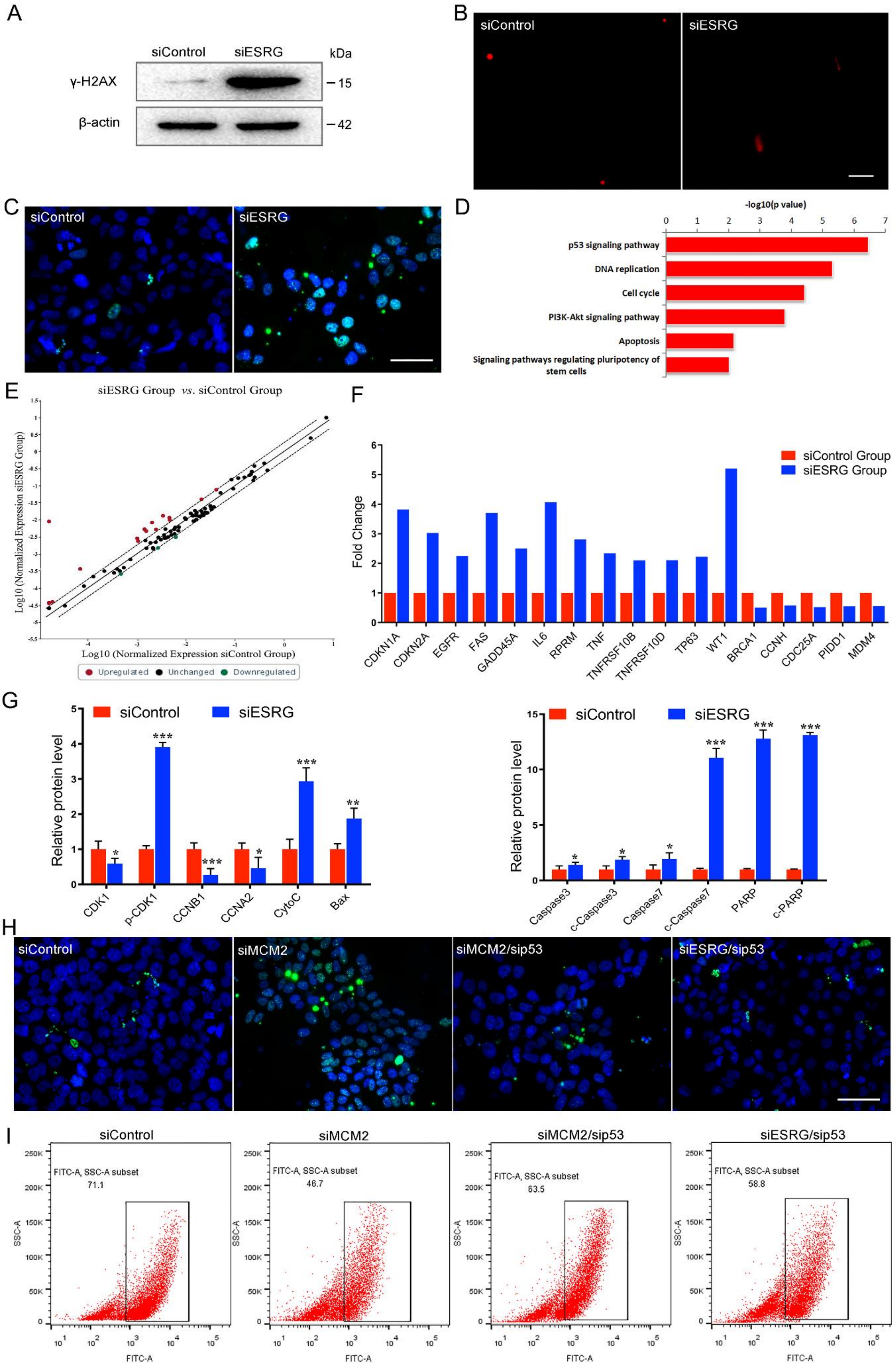
A
siControl
siESRG
kDa
γ-H2AX
15
β-actin
42
B
siControl
siESRG
C
siControl
siESRG
D
-log10(p value)
p53 signaling pathway
DNA replication
Cell cycle
PI3K-Akt signaling pathway
Apoptosis
Signaling pathways regulating pluripotency of stem cells
E
siESRG Group vs. siControl Group
Log10 (Normalized Expression siESRG Group)
Log10 (Normalized Expression siControl Group)
Upregulated
Unchanged
Downregulated
F
Fold Change
siControl Group
siESRG Group
CDKN1A
CDKN2A
EGFR
FAS
GADD45A
IL6
RPRM
TNF
TNFRSF10B
TNFRSF10D
TP63
WT1
BRCA1
CCNH
CDC25A
PIDD1
MDM4
G
siControl
siESRG
Relative protein level
CDK1
p-CDK1
CCNB1
CCNA2
CytoC
Bax
Caspase3
c-Caspase3
Caspase7
c-Caspase7
PARP
c-PARP
H
siControl
siMCM2
siMCM2/sip53
siESRG/sip53
I
siControl
siMCM2
siMCM2/sip53
siESRG/sip53
FITC-A, SSC-A subset
71.1
46.7
63.5
58.8
SSC-A
FITC-A

Fig. S6. *ESRG*-MCM2 functions in hPSCs by suppressing p53 signaling pathway.

(A) Western blot was performed to analyze the protein levels of γ-H2AX in RC1-hiPSCs treated with siControl and si*ESRG*. **(B)** Representative pictures of comet assay performed 48 h after RC1-hiPSCs treated with siControl and si*ESRG*. Scale bar, 200 μm. **(C)** γH2AX expression was detected by immunofluorescence staining after *ESRG* knockdown in RC1-hiPSCs. Scale bar, 100 μm. **(D)** The top KEGG pathway enrichments for differentially expressed genes between si*ESRG* and siControl by mRNA microarray. **(E)** The expression levels of 84 genes related to p53-mediated signal transduction were evaluated using a RT Profiler PCR Array in *ESRG* knockdown and control groups. Scatter plots indicate at least a two-fold up-regulated (red) or down-regulated (green) gene in the *ESRG* knockdown groups relative to the control group. **(F)** The fold changes in expression of these genes in the *ESRG* knockdown groups relative to the control group are shown in the column chart. **(G)** Quantification of proteins and statistical analyses of Fig. 6H. **(H-I)** H9 hESCs were transfected with p53 siRNA followed by MCM2/*ESRG* knockdown. γ-H2AX protein expression was detected by immunofluorescence staining (H) and EdU assay was performed by flow cytometry (I). Scale bar, 100 μm. Data are presented as mean ± SD. $*P < 0.05$, $**P < 0.01$, $***P < 0.001$ (two-tailed Student's *t* test).

Table S1. RNA-pulldown identified *ESRG* binding Proteins.

Protein names	Descriptions
**ACO2**	Aconitate hydratase, mitochondrial
**APEH**	Acylamino-acid-releasing enzyme
**ASNS**	Asparagine synthetase [glutamine-hydrolyzing]
**BSG**	Basigin
**CASP14**	Caspase-14
**CBR1**	Carbonyl reductase [NADPH] 1
**CBS**	Cystathionine beta-synthase
**CCT7**	T-complex protein 1 subunit eta
**CDC37**	Hsp90 co-chaperone Cdc37
**CSTA**	Cystatin-A
**EIF2S1**	Eukaryotic translation initiation factor 2 subunit 1
**EIF3C**	Eukaryotic translation initiation factor 3 subunit C
**ENO2**	Gamma-enolase
**FLII**	Protein flightless-1 homolog
**FSCN1**	Fascin
**GANAB**	Neutral alpha-glucosidase AB
**GART**	Trifunctional purine biosynthetic protein adenosine-3
**GMPS**	GMP synthase [glutamine-hydrolyzing]
**GOT1**	Aspartate aminotransferase, cytoplasmic
**GSPT2**	Eukaryotic peptide chain release factor GTP-binding subunit ERF3B
**GSTP1**	Glutathione S-transferase P
**H2AFJ**	Histone H2A.J
**HBA1**	Hemoglobin subunit alpha
**HMGCS1**	Hydroxymethylglutaryl-CoA synthase, cytoplasmic
**HNRNPA1**	Heterogeneous nuclear ribonucleoprotein A1

**HSPA1L**	Heat shock 70 kDa protein 1-like
**HSPH1**	Heat shock protein 105 kDa
**IPO5**	Importin-5
**LAP3**	Cytosol aminopeptidase
**LARS**	Leucine--tRNA ligase, cytoplasmic
**LMNB1**	Lamin-B1
**LRPPRC**	Leucine-rich PPR motif-containing protein, mitochondrial
**MCCC1**	Methylcrotonoyl-CoA carboxylase subunit alpha, mitochondrial
**MCM2**	DNA replication licensing factor MCM2
**MTHFD1**	C-1-tetrahydrofolate synthase, cytoplasmic
**NACA**	Nascent polypeptide-associated complex subunit alpha, muscle-specific form
**NME2**	Nucleoside diphosphate kinase B
**NPM1**	Nucleophosmin 1
**P4HB**	Protein disulfide-isomerase
**PC**	Pyruvate carboxylase, mitochondrial
**PCMT1**	Protein-L-isoaspartate(D-aspartate) O-methyltransferase
**PDIA6**	Protein disulfide-isomerase A6
**PEBP1**	Phosphatidylethanolamine-binding protein 1
**PHB2**	Prohibitin-2
**PMPCB**	Mitochondrial-processing peptidase subunit beta
**PODXL**	Podocalyxin
**PSMB5**	Proteasome subunit beta type-5
**PSMD11**	26S proteasome non-ATPase regulatory subunit 11
**RAB8A**	Ras-related protein Rab-8A
**RANGAP1**	Ran GTPase-activating protein 1
**RRBP1**	Ribosome-binding protein 1
**RTN4**	Reticulon-4
**RUVBL1**	RuvB-like 1
**SDHA**	Succinate dehydrogenase [ubiquinone] flavoprotein subunit, mitochondrial
**SEPT9**	Septin-9

**SF1**	Splicing factor 1
**SF3A1**	Splicing factor 3A subunit 1
**SF3B3**	Splicing factor 3B subunit 3
**SH3GL2**	Endophilin-A1
**SLC3A2**	4F2 cell-surface antigen heavy chain
**ST13**	Hsc70-interacting protein
**STIP1**	Stress-induced-phosphoprotein 1
**TCP1**	T-complex protein 1 subunit alpha
**TMED10**	Transmembrane emp24 domain-containing protein 10
**TUBA1C**	Tubulin alpha-1C chain
**TXNDC5**	Thioredoxin domain-containing protein 5
**UBE2NL**	Putative ubiquitin-conjugating enzyme E2 N-like
**UBQLN2**	Ubiquilin-2
**UQCRC1**	Cytochrome b-c1 complex subunit 1, mitochondrial
**USO1**	General vesicular transport factor p115
**VIL1**	Villin-1
**WDR1**	WD repeat-containing protein 1

Table S2. Gene names of enriched pathways in the differentially altered p53-target genes in hPSCs upon *ESRG* knockdown.

Term	*P*-value	Genes
**p53 signaling pathway**	3.48*E*-07	BID, STEAP3, ZMAT3, CHEK1, SFN, SESN2, CCNG2, CCNE2, CCNE1, CASP9, SERPINE1, RCHY1, THBS1, LOC651610, CDK1, CYCS, CDK6, ATM, CCNB1, CDKN1A, PPM1D, TNFRSF10B, RRM2, BAX, GADD45G, DDB2, MDM4, GADD45B, PERP, GADD45A
**DNA replication**	4.91*E*-06	SSBP1, LIG1, POLE, POLA1, POLA2, MCM2, MCM3, MCM4, RNASEH2C, MCM5, MCM6, RPA1, RFC5, POLD3, DNA2, RFC3, POLE2, RFC2, PRIM2, PCNA, FEN1
**Cell cycle**	3.82*E*-05	CDC14B, PRKDC, CHEK1, SFN, CCNE2, CCNE1, CDC45, ORC4L, CDKN2D, CCNA1, MYC, CUL1, STAG1, TFDP1, LOC651610, CDK1, CDC6, RBL1, ANAPC4, CDK6, ESPL1, MCM2, MCM3, MCM4, ORC1L, MCM5, CDC25A, WEE1, ATM, MCM6, CCNB1, CDKN1C, CDKN1A, CDKN1B, GADD45G, PCNA, LOC731751, ORC5L, ANAPC7, GADD45B, GADD45A

Table S3. Primer sequences for qPCR.

Genes	Sequences (5′-3′) Forward	Sequences (3′-5′) Reverse
GAPDH	GATGACATCAAGAAGGTGGTGA	GTCTACATGGCAACTGTGAGGA
ESRG	CTCCCCAGAACATCTCCAGAA	TCTGGACATTGTCCTTCCAACT
Oct3/4	TTCAGCCAAACGACCATCTG	CACGAGGGTTTCTGCTTTGC
Cripto	TACCTGGCCTTCAGAGAT	CCAGCATTTACACAGGGAACAC
FoxD3	CTAGTGAAGCCGCCTTACTCGTA	GAAGCAGTCGTTGAGTGAGAGGTT
Tert1	TGTGCACCAACATCTACAAG	GCGTTCTTGGCTTTCAGGAT
U6	CTCGCTTCGGCAGCACA	AACGCTTCACGAATTTGCGT
Nanog	TGAACCTCAGCTACAAACAG	TGGTGGTAGGAAGAGTAAAG
LIN28A	CGGGCATCTGTAAGTGGTTC	CAGACCCTTGGCTGACTTCT
LIN28B	CATCTCCATGATAAACCGAGAGG	GTTACCCGTATTGACTCAAGGC
DESMIN	GCACGCCCTCCTCCTAC	GCAGCTCCACCTTCTCGT
JMJD5	GGAGCAGTTTTTGGTTCCAGG	GGCTCATTCACGATGTATTTGC
Wee1	CCTGGGTAGCTCTTTCTCG	TTGCGGAAGGTCTTGTGT
GATA4	AAGCCCAAGAACCTGAATAAATC	TGGCGTTGCTGGAGTTG
GATA6	CCATGACTCCAACTTCCACC	ACGGAGGACGTGACTTCGGC
OPN	GCCGAGGTGATAGTGTGGTT	TGAGGTGATGTCCTCGTCTG
AFP	TTGGGCTGCTCGCTATG	TTTGTAACTGTTGCTGCCTTTG
SOX17	CGCCGAGTTGAGCAAGA	TTCAGCCGCTTCACCTG
GATA2	CAGACGACAACCACCACCTTATG	TGGTCAGTGGCCTGTTAACATTG
NESTIN	TGCGGGCTACTGAAAAGTTC	TGTAGGCCCTGTTTCTCCTG
SCL	CGGCAGCGGGTTCTTTG	CCCGGCTGTTGGTGAAGATAC

HAND1	TCAGCCTTGCCCGGACTCTC	AGGTTCATGTTGGAGCGGCTAC
MSX1	CCAGAAGATGCGCTCGTCAAAG	CGGCTTACGGTTCGTCTTGTG
LEFTY	CTGGACAGGGCCGACATG	GGCCACCTCTCGGAAGCT
SOX1	ATACGTTTATTTCAGCAGCCTTAGG	TCCAGGACAAGGAAGGGTGTT
PAX6	GGCTAGCGAAAAGCAACAGA	TGGTATTCTCTCCCCCTCCT
CGα	CTTTCTGCATGTTCTCCATTC	GTGGACTCTGAGGTGACGT
CGβ	TCACCGTCAACACCACCATC	AGAGTGCACATTGACAGCTG
p53	AAGTCTGTGACTTGCACGTACTCC	GTCATGTGCTGTGACTGCTTGTAG
p21	ACCTGGAGACTCTCAGGGTCG	TTAGGGCTTCCTCTTGGAGAAGAT
GADD45A	GGATGCCCTGGAGGAAGTGCT	GGCAGGATCCTTCCATTGAGATGAATG
DR5	CTCCTGCAAATATGGACAGGACTA	TTAGCTCCACTTCACCTGAATCAC
14-3-3σ	TGACGACAAGAAGCGCATCAT	GTAGTGGAAGACGGAAAAGTTCA

Table S4. Antibodies used in this study.

REAGENT or RESOURCE	SOURCE	IDENTIFIER
Anti-OCT4	Abcam	Cat# ab181577; RRID: AB_2687916
Anti-NANOG	Abcam	Cat# ab109250; RRID: AB_10863442
Anti-gamma H2A.X(phospho S139)	Abcam	Cat# ab81299; RRID: AB_1640564
Anti-MCM2	Proteintech	Cat# 10513-1-AP; RRID: AB_2142131
Anti-Flag Tag	Sigma-Aldrich	Cat# F1804 RRID: AB_262044
Anti-p53(DO-1)	Santa Cruz Biotechnology	Cat# sc-126; RRID:AB_628082
Anti-p21	Cell Signaling Technology	Cat# 2947 RRID: AB_823586
Anti-DR5	Proteintech	Cat#15497-1-AP; RRID: AB_2240702
Anti-14-3-3 zeta/delta (D7H5)	Cell Signaling Technology	Cat# 7413; RRID: AB_10950820
Anti-FAS	Abcam	Cat# ab82419; RRID: AB_1658628

Anti-SSEA4	Abcam	Cat# ab16287; RRID: AB_778073
Anti-TRA-1-60	Santa Cruz Biotechnology	Cat# sc-21705; RRID: AB_628385
Anti-Ub(P4D1)	Santa Cruz Biotechnology	Cat# sc-8017; RRID: AB_2762364
Anti-Cleaved Caspase3 (Asp175)	Cell Signaling Technology	Cat# 9664; RRID: AB_2070042
Anti-Caspase3	Cell Signaling Technology	Cat# 9665; RRID: AB_2069872
Anti-Cleaved Caspase7 (Asp198)	Cell Signaling Technology	Cat# 8438; RRID: AB_11178377:
Anti-Caspase7	Cell Signaling Technology	Cat# 12827; RRID: AB_2687912
Anti-CDK1	Abcam	Cat# ab18; RRID: AB_2074906
Anti-CDK1 (phospho Y15)	Abcam	Cat# ab47594 RRID: AB_869073
Anti-Cleaved PARP	Cell Signaling Technology	Cat# 5625; RRID: AB_10699459
Anti- PARP	Cell Signaling Technology	Cat# 9542; RRID: AB_2160739

Anti-Phospho p53 (Ser15)	Cell Signaling Technology	Cat# 9284; RRID: AB_331464
Anti-Wee1(D10D2)	Cell Signaling Technology	Cat# 13084; RRID: AB_2713924
Anti-GADD45Aα(D17E8)	Cell Signaling Technology	Cat# 4632; RRID: AB_10544538
Anti-LIN28A	Abcam	Cat# ab46020; RRID: AB_776033
Anti-TetR	Novus Biologicals	Cat# NB600-234; RRID: AB_10001361
Anti-Histone H3	Proteintech	Cat# 17168-1-AP; RRID: AB_2716755
Anti-Cyclin B1 (CCNB1)	Cell Signaling Technology	Cat# 4138; RRID: AB_2072132
Anti-Cyclin A2 (CCNA2)	Proteintech	Cat# 18202-1-AP; RRID: AB_10597084
Anti-BAX	Proteintech	Cat# 50599-2-Ig; RRID: AB_2061561
Anti-Cytochrome C (Cyto-C)	Thermo Fisher Scientific	Cat# MA5-11674; RRID: AB_10985701
Anti-ATM	Cell Signaling Technology	Cat# 2873; RRID: AB_2062659

Anti-ATM (phospho S1981)	Abcam	Cat# ab81292; RRID: AB_1640207
Anti-TRAIP	Proteintech	Cat# 10332-1-AP; RRID: AB_10638481
Anti-GAPDH	Abcam	Cat# ab181602; RRID: AB_2630358
Anti-beta Actin antibody	Sigma	Cat# ab8227; RRID: AB_2305186
Donkey anti-Mouse IgG (H+L) Secondary Antibody, Alexa Fluor Plus 594	Thermo Fisher Scientific	Cat# A-21203; RRID: AB_2535789
Donkey anti-Rabbit IgG (H+L) Secondary Antibody, Alexa Fluor 488	Thermo Fisher Scientific	Cat# A-21206; RRID: AB_2535792
